# Design of amyloidogenic peptide traps

**DOI:** 10.1038/s41589-024-01578-5

**Published:** 2024-03-19

**Authors:** Danny D. Sahtoe, Ewa A. Andrzejewska, Hannah L. Han, Enrico Rennella, Matthias M. Schneider, Georg Meisl, Maggie Ahlrichs, Justin Decarreau, Hannah Nguyen, Alex Kang, Paul Levine, Mila Lamb, Xinting Li, Asim K. Bera, Lewis E. Kay, Tuomas P. J. Knowles, David Baker

**Affiliations:** 1 Department of Biochemistry, University of Washington, Seattle, WA USA; 2 Institute for Protein Design, University of Washington, Seattle, WA USA; 3 HHMI, University of Washington, Seattle, WA USA; 4 Yusuf Hamied Department of Chemistry, University of Cambridge, Cambridge, UK; 5 Department of Molecular Genetics, University of Toronto, Toronto, Ontario Canada; 6 Department of Biochemistry, University of Toronto, Toronto, Ontario Canada; 7 Department of Chemistry, University of Toronto, Toronto, Ontario Canada; 8 Program in Molecular Medicine, The Hospital for Sick Children Research Institute, Toronto, Ontario Canada; 9 Cavendish Laboratory, University of Cambridge, Cambridge, UK; 10 Present Address: Hubrecht Institute, Utrecht, the Netherlands

**Keywords:** Protein design, Biologics, Molecular neuroscience

## Abstract

Segments of proteins with high β-strand propensity can self-associate to form amyloid fibrils implicated in many diseases. We describe a general approach to bind such segments in β-strand and β-hairpin conformations using de novo designed scaffolds that contain deep peptide-binding clefts. The designs bind their cognate peptides in vitro with nanomolar affinities. The crystal structure of a designed protein−peptide complex is close to the design model, and NMR characterization reveals how the peptide-binding cleft is protected in the apo state. We use the approach to design binders to the amyloid-forming proteins transthyretin, tau, serum amyloid A1 and amyloid β_1−42_ (Aβ42). The Aβ binders block the assembly of Aβ fibrils as effectively as the most potent of the clinically tested antibodies to date and protect cells from toxic Aβ42 species.

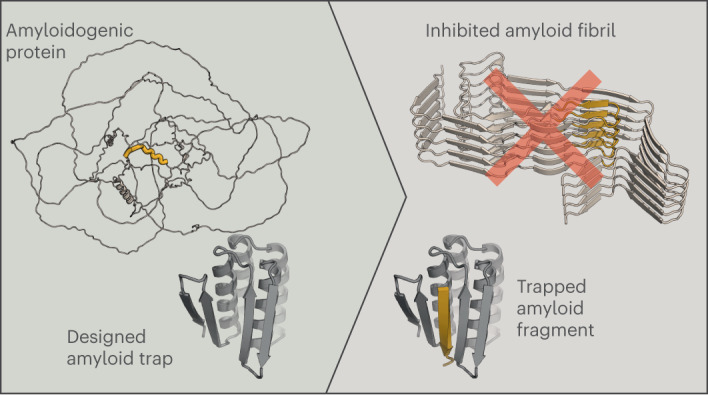

## Abstract (web summary)

An approach to design proteins that can capture amyloidogenic protein regions present in, for example, tau and Aβ42 has now been developed. These designer proteins can inhibit the formation of pathogenic amyloid fibrils and protect cells from toxic species.

## Main

Many proteins contain segments that become ordered only upon self-association or binding of an interaction partner^1–3^. A particularly interesting example of such disorder-to-order transitions is the amyloidogenic sequences that are found in proteins such as amyloid-β_1−42_, (Aβ42), tau and serum amyloid A1. These regions can aggregate into amyloid fibrils via strand−strand interactions and are associated with amyloidosis and associated diseases both inside and outside the central nervous system^4–9^. Although the correlation between amyloid formation and neurodegenerative disease remains incompletely understood, designed binders to amyloid-forming segments of these proteins could have utility as diagnostics, therapeutics and research tools. However, the disordered nature of amyloidogenic protein segments complicates the generation of amyloid inhibitors. This complication can be circumnavigated by generating binders against amyloid fibrils for which a number of three-dimensional (3D) structures have been determined^10–15^. This structure-based approach has yielded amyloid binders and peptides that ‘cap’ amyloid fibril ends and retard fibril nucleation and growth^16,17^. When structural information is not available, for instance when targeting disordered amyloidogenic protein segments, library selection-based methods can be used. Through these approaches, antibody and affibody molecules have been evolved^18–22^ that inhibit amyloid fibril formation, but it is difficult to target specific regions and specific conformational states using these approaches. The ability to computationally design binders to any disordered amyloidogenic segment and bypass library selection methods would be of considerable use in biotechnology and biomedical research by facilitating the development of new molecules with user-defined properties. While there have been considerable advances in computational protein design, the multiplicity of conformations complicates the design of binders to disordered amyloidogenic protein segments, and the computational design of binders to amyloid-forming segments of proteins remains an outstanding challenge.

## Results

### Design strategy for extended-strand binders

**Fig. 1:**
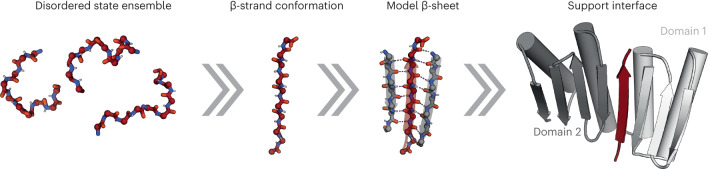
Design approach for binding disordered protein fragments. Intrinsically disordered regions of proteins and peptides have large conformational freedom but may be forced into predefined conformations such as β-strands that can be efficiently targeted by modeling a β-sheet that hydrogen bonds with the peptide. The interaction is stabilized by additional secondary structure elements supported by a two-domain single-chain protein flanking each side of the target peptide.

We reasoned that the challenge of binding disordered amyloidogenic peptides could be overcome by taking advantage of their β-strand-forming propensity. Binding of peptides in the β-strand conformation has been observed in nature^23,24^, and the regularity of the β secondary structure has been exploited to computationally design interactions between pairs of folded proteins, including homodimers, binders to target proteins with exposed β-strands and nanoscale multisubunit hetero-oligomers^25–28^. To design binders to peptides in extended β-strand conformations, we sought to create scaffolds that could provide β-strand pairing interactions to all the backbone amide and carbonyl atoms of the peptide, such that the peptide strand complements a β-sheet on the scaffold (Fig. 1). Starting from FoldIt designed proteins with mixed α/β topology^29^, we designed additional strands and helices to create scaffolds with a single central β-strand missing from an extended β-sheet. The sheet is buttressed by α-helices that pack on one another to support the structure in the absence of the bound peptide (Fig. 1, Supplementary Fig. 1 and Methods). Rosetta combinatorial sequence design calculations were then used to optimize the sequences of both the scaffold and the peptide for high-affinity binding (we reasoned that such ‘two-sided’ designs would be an easier starting point than ‘one-sided’ designs against amyloid-forming peptides where only the sequences of the binders are allowed to be optimized). A total of 116 designs with favorable interaction energy, together with few unsatisfied buried polar atoms and high shape complementarity, for which Rosetta ab initio structure predictions were close to the designed scaffold and complex structures, were selected for experimental characterization.

### Characterization of designed peptide−binder pairs

The selected designs, without their cognate peptides, were encoded in synthetic genes with an N-terminal polyhistidine affinity tag, expressed in *Escherichia coli*, and purified using immobilized metal affinity chromatography (IMAC) with nickel followed by size exclusion chromatography (SEC). Despite the absence of the peptide, 36% of the designs expressed well and were monodisperse in SEC. Bicistronic vectors were generated for each of the monodisperse designs; the first cistron encoded superfolding green fluorescent protein (sfGFP) fused at its C terminus to the designed peptide, and the second cistron encoded the polyhistidine-tagged designed binder. After expression of the bicistronic constructs, binding of the GFP−peptide fusion to the His-tagged binder was assessed by SDS−PAGE following purification by IMAC and SEC. About half of these designs formed a complex in SEC.

**Fig. 2:**
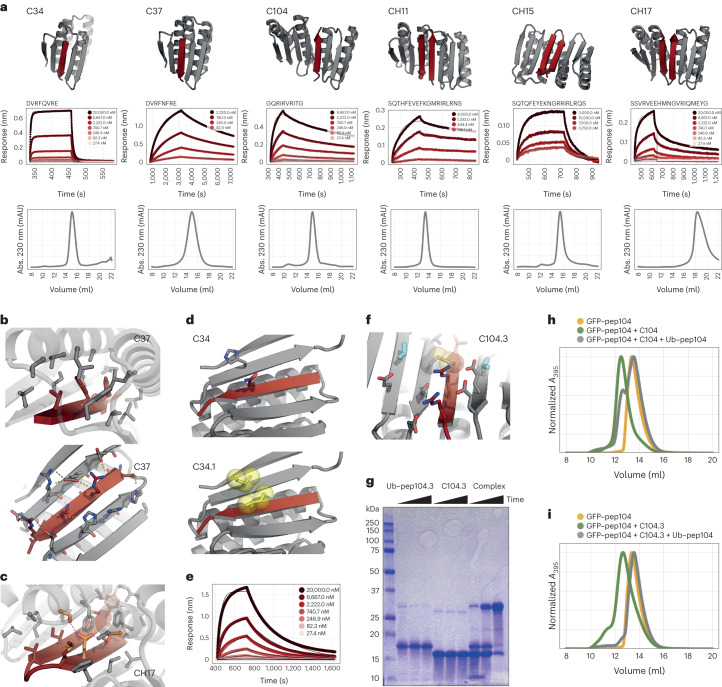
Characterization of designed peptide binders. **a**, Design models for peptide binders (binder, gray; peptide, dark red). BLI traces with kinetic fits and SEC (S75 Increase 10/300) chromatograms of the purified binders are shown below the corresponding models. mAU, milli absorbance units. **b**, Detailed views of the solvent-exposed interface (bottom) and the buried interface (top) of C37. C-α atoms as spheres. **c**, Detailed view of the buried part of the interface of hairpin binder CH17 with the designed hydrogen-bond network depicted in orange sticks. **d**, Models of parent design C34 (top) and C34.1 (bottom) where a hydrophobic interaction pair (yellow sticks/spheres) is introduced to improve affinity. **e**, BLI traces of C34.1 binding to its peptide immobilized on biosensors. **f**, View of the designed interface disulfide on C104.3 (disulfide in spheres and sticks; additional redesigned residues in cyan). **g**, Representative nonreducing SDS−PAGE gel showing disulfide formation (time points of 0 min, 90 min and overnight). The experiment was reproduced twice with two independent protein preparations. Ub, ubiquitin. **h**, SEC traces of preformed noncovalent C104 complex + GFP−pep104. **i**, SEC traces of preformed covalent disulfide-linked C104.3 complex + GFP−pep104. Source data

**Extended Data Fig. 1:**
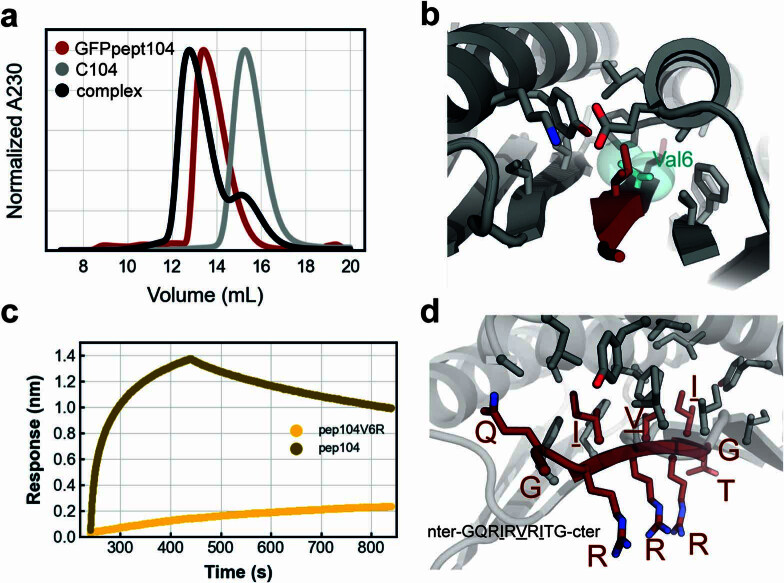
Designed peptide binder controls. **a**, SEC binding assay showing that a fusion protein between GFP and 104 peptide binds to the C104 design on a S75 increase 10/300. **b**, Close-up view of the buried part of the C104 interface with Val6 shown in cyan sticks and spheres. Binder in gray and peptide in dark red. **c**, Biolayer interferometry trace of C104 binding to base peptide 104 and to a peptide with a V6R substitution. **d**, Interface close up view of C104 highlighting the hydrophobic-hydrophilic pattern of the peptide. Buried residues single letter amino acid identifiers are underlined. Source data

The six designs (Fig. 2a, Supplementary Fig. 2, Supplementary Table 1 and Source data) that were the most well expressed, soluble and monodisperse by SEC were tested for their ability to bind to their designed peptide targets using biolayer interferometry (BLI), immobilizing chemically synthesized biotinylated peptides on streptavidin sensors and then dipping them into a solution with the purified designed binding partner. The interaction kinetics ranged from 10^4^ M^−1^ s^−1^ to 10^2^ M^−1^ s^−1^ for association and between 0.17 s^−1^ and 10^–4^ s^−1^ for dissociation (Supplementary Table 2). The equilibrium dissociation constant *K*_d_ ranged from 44 μM to 150 nM with no clear distinction between single-strand and hairpin binders (Fig. 2a and Supplementary Table 2). For design C104, we confirmed binding in an orthogonal SEC binding assay (Extended Data Fig. 1a). Single-amino acid substitution of the buried residue Val6 in the peptide of C104 to arginine completely disrupted binding in BLI, suggesting that the designed binding mode is recapitulated (Extended Data Fig. 1b, c).

**Extended Data Fig. 2:**
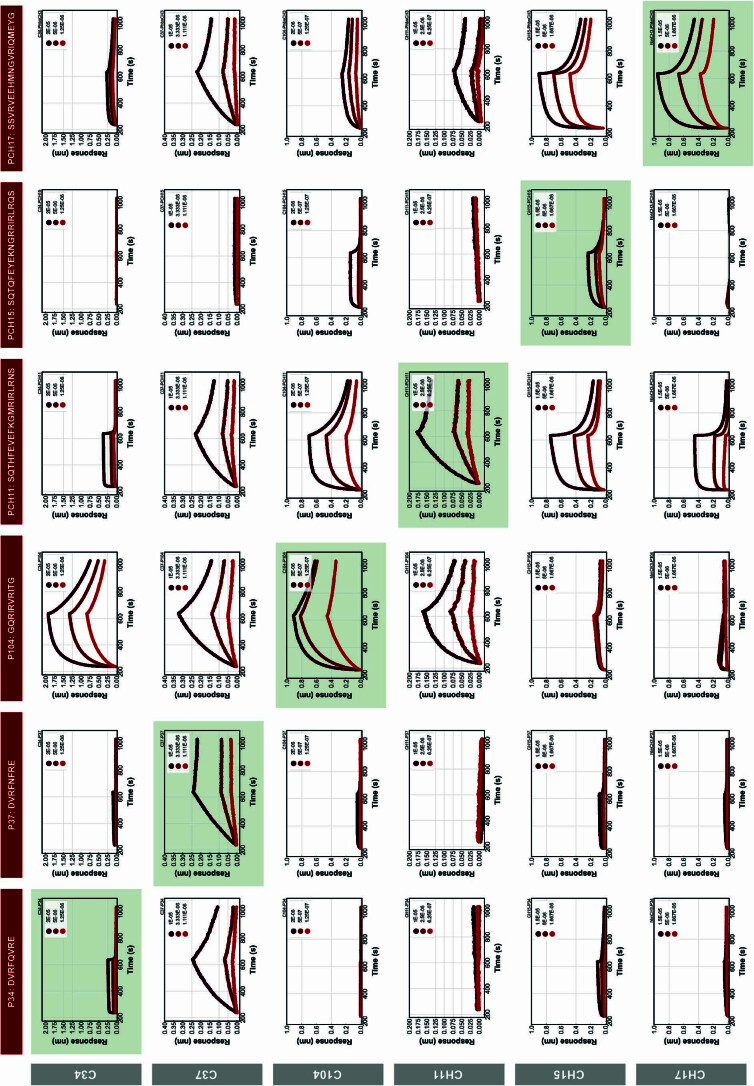
Specificity profile of peptide binder designs in BLI. Peptides were immobilized onto octet biosensors at equal densities and incubated with all designs in separate experiments at three different binder concentrations. The on-target interactions are indicated with a light green background. The experiment was done for each different peptide from the base designs (Fig. 2a). Source data

The designed peptides are amphipathic with an alternating hydrophilic−hydrophobic side chain pattern (Extended Data Fig. 1d). Beyond the backbone β-strand hydrogen bonding, the peptide−binder interaction consists of somewhat separable solvent-exposed and solvent-shielded interfaces. The solvent-inaccessible part of the interface consists primarily of the hydrophobic residues that closely pack against the hydrophobic core of the binder and drive the association between peptide and binder (Fig. 2b, top). In design CH17, these interactions are accompanied by designed buried hydrogen-bond networks (Fig. 2c)^30^. The solvent-exposed portion of the interface (Fig. 2b, bottom) is composed primarily of salt bridges and hydrogen bonds that likely make less of a contribution to the overall interface energy because of competition with water. Because the hydrophobic−hydrophilic patterning is shared among the designed peptides, not all binder designs can fully discriminate between cognate and noncognate peptides, enabling them to sequester a broad range of peptides that have similar physicochemical properties (Extended Data Fig. 2). Design CH17 that contains a buried hydrogen-bond network is more selective for its cognate peptide, likely because binding of a noncomplementary peptide would bury polar residues not compatible with this network (Extended Data Fig. 2).

**Extended Data Fig. 3:**
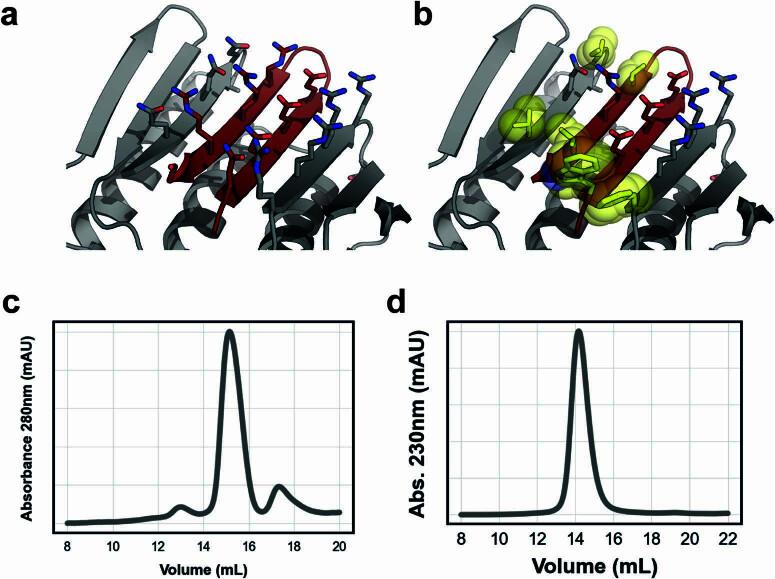
Computational affinity maturation by introducing solvent exposed hydrophobic interaction pairs. **a**, View of the solvent exposed interface of CH15 (binder gray, peptide dark red). **b**, View of the redesigned CH15.1 interface. Hydrophobic interaction pairs introduced to the base CH15 scaffold to improve affinity are highlighted in yellow sticks and spheres. Superdex 75 Increase 10/300 GL SEC traces of purified C34.1. **(c)** and CH15.1 **(d)**. Source data

We explored the possibility of increasing peptide binding affinity by introducing hydrophobic interaction pairs across solvent-exposed parts of the interface using a combinatorial side chain design in Rosetta. The introduction of an exposed hydrophobic interaction pair in design C34.1 improved the *K*_d_ by sixfold to 2 μM from 12 μM for the parent design C34 (Fig. 2d,e and Supplementary Table 2). In CH15.1, we introduced three hydrophobic interaction pairs that when combined led to a 400-fold improvement of the *K*_d_ from 40 μM for the parent CH15 design to 100 nM (Fig. 4f, Extended Data Fig. 3a,b and Supplementary Table 2). The modified designs remained monomeric, indicating that these surface substitutions are generally well tolerated (Extended Data Fig. 3c,d).

**Extended Data Fig. 4:**
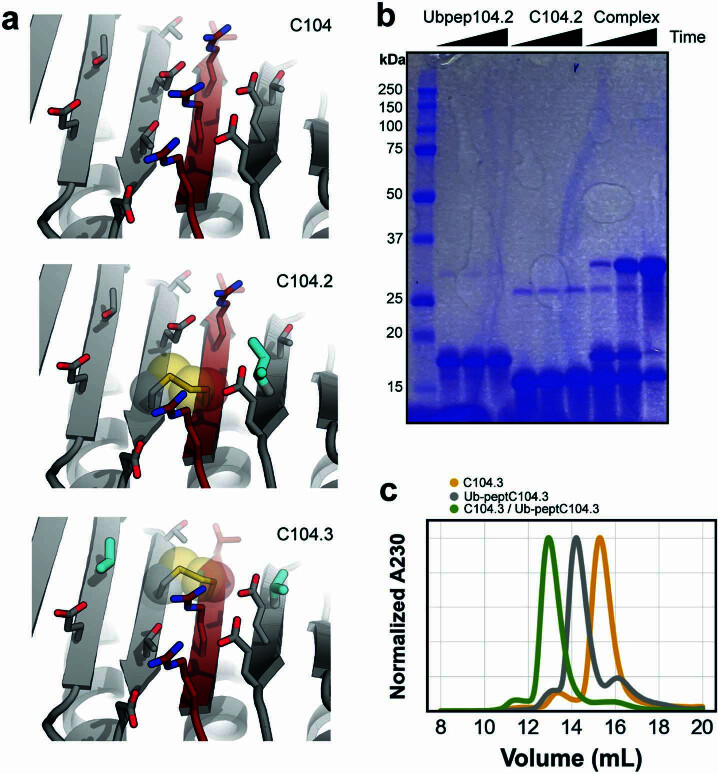
Disulfide functionalization of C104. **a**, Close-up of C104 surface exposed interface (top) and of the disulfide bridge variants C104.2 (middle) and C104.3 (bottom). Disulfide bonds are highlighted with spheres while additional redesigned residues to optimally accommodate the disulfide bridges are highlighted in cyan thicker sticks. Designed binder in gray and peptide in dark red. **b**, Representative coommassie stained non-reducing SDS-PAGE gel monitoring disulfide bridge formation of C104.2. Time points are t=0, t=90min and t=overnight. Experiment was reproduced twice with 2 independent protein preparations **c**, Superdex 75 increase 10/300 GL SEC binding assay confirming that the cysteine containing peptide of C104.3 fused to ubiquitin can bind to its designed cysteine containing binding partner C104.3. Source data

Disulfide functionalization could enable redox control of binding activity for a variety of biotechnological applications. We searched for positions that could host a disulfide bridge across the interface of C104 using the disulfidize mover in Rosetta^31,32^ and found several positions where low-energy disulfides could be modeled (Fig. 2f and Extended Data Fig. 4a). For designs C104.2 and C104.3, we confirmed through nonreducing SDS−PAGE analysis that disulfides formed (Fig. 2g and Extended Data Fig. 4a,b). For C104.3, this result was validated in an SEC subunit exchange experiment where we first reconstituted the noncovalent complex between C104 and its peptide fused to the C terminus of ubiquitin, as well as the disulfide-linked complex between C104.3 and its cysteine-containing peptide fused to the C terminus of ubiquitin. When the preformed noncovalent complex was mixed with C104 peptide fused to GFP (GFP−pep104) and followed by SEC, GFP−pep104 co-eluted with C104 (as indicated by the absorbance at 395 nm), indicating that GFP−pep104 could exchange with the ubiquitin−peptide fusion to bind C104 (Fig. 2h). In contrast, the peptide in the covalent C104.3 complex could not be outcompeted when it was mixed with GFP−pep104, presumably due to the disulfide bridge (Fig. 2i and Extended Data Fig. 4c).

**Fig. 3:**
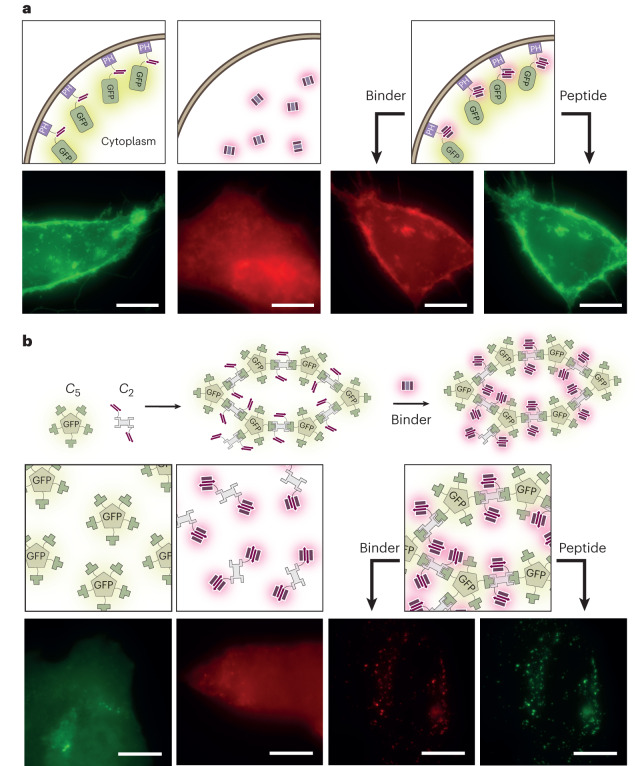
Designed peptide−binder pairs function in mammalian cells. **a**, Representative fluorescence microscopy images of mScartlet−CH15.1 localization to membranes in HeLa cells. Scale bars, 10 μm. PH, Pleckstrin homology domain. **b**, Representative fluorescence microscopy images of mScartlet−CH15.1 localizing to designed intracellular GFP-positive protein puncta in HeLa cells. Scale bars, 10 μm. Results were reproduced in two independent experiments. Source data

To examine the functionality of the designs in mammalian cells, we transfected HeLa cells with a construct with the CH15.1 peptide fused to the N terminus of GFP and to the C terminus of the phospholipase-C Pleckstrin homology domain that binds phosphatidylinositol 4,5-bisphosphate at the outer plasma membrane^33^. Fluorescence microscopy analysis showed that the plasma membranes of the transfected cells were labeled green. When cells were additionally transfected with mScarlet-labeled CH15.1 binder, GFP and mScarlet colocalized at the plasma membrane, indicating binding (Fig. 3a and Supplementary Fig. 3a,b). In control cells that were transfected with only mScarlet−CH15.1, or with mScarlet−CH15.1 and a mutant peptide with a substitution intended to disrupt binding, no colocalization was observed, indicating that the interaction takes place through the designed interface (Fig. 3a and Supplementary Fig. 3c).

In a second cell-based experiment, we tested whether the binder−peptide interaction could localize to intracellular two-component protein puncta. We generated the puncta using the LHD hetero-oligomer system that consists of de novo designed protein building blocks that can be assembled into a large variety of multiprotein complexes^28^. The first construct was a homopentamer fused to GFP and one half of a designed LHD-heterodimer, and the second component, a pseudo-*C*_2_-symmetric design that presented two copies of the other half of the designed heterodimer and was also fused to the peptide of CH15.1 (Fig. 3b). When the homopentamer was expressed in HeLa cells, we observed a diffuse GFP distribution. Upon coexpression of the second component, a protein network was formed through the designed LHD heterodimer interfaces as observed by the formation of GFP puncta (Fig. 3b). When mScarlet-tagged CH15.1 binder was present, it was recruited to the puncta, whereas in control experiments where the puncta did not form or where the mutant peptide was expressed the mScarlet−CH15.1 binder was distributed uniformly throughout the cell, indicating that the peptide−binder pair can specifically associate within the crowded environment of the cell (Fig. 3b and Supplementary Fig. 3d–g).

**Extended Data Fig. 5:**
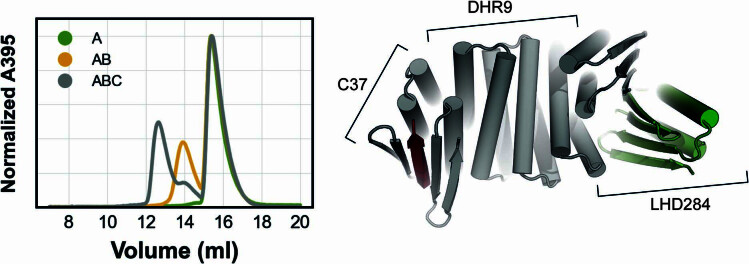
Incorporation of C37 into LHD hetero-oligomer system. Design C37 was rigidly fused to LHD284B_DHR9 (right) creating a single chain protein with two interfaces capable of binding the peptide of C37 and the designed binding partner of LHD284B_DHR9, LHD284A_DHR82. We validated the assembly of this ternary complex in a SEC binding assay on a S200 increase 10/300 GL. A: GFP-peptC37, AB: GFP-peptC37 + LHD284B_DHR9, ABC: GFP-peptC37 + LHD284B_DHR9 + LHD284A_DHR82. Absorbance at 395 nm of the GFP-peptC37 was monitored to assess binding. Source data

Small peptides are useful as affinity tags to bind and localize tagged protein partners into larger molecular assemblies. In nature, this method of protein recruitment is commonly used to regulate various cellular processes in a dynamic fashion. To demonstrate the utility of our designs for such applications and also for use in novel customizable protein materials, we rigidly fused binder C37 to one half of a LHD heterodimer (LHD284B9)^28^. Fusion creates single-chain proteins with two different interfaces: one peptide-binding interface and one LHD heterodimerization interface. Mixing the GFP-tagged peptide of C37 with C37LHD284B9 creates a heterodimer, which can be built upon by the addition of LHD284A82 to form a heterotrimer (Extended Data Fig. 5).

### Structural analysis of designed peptide−binder complexes

**Fig. 4:**
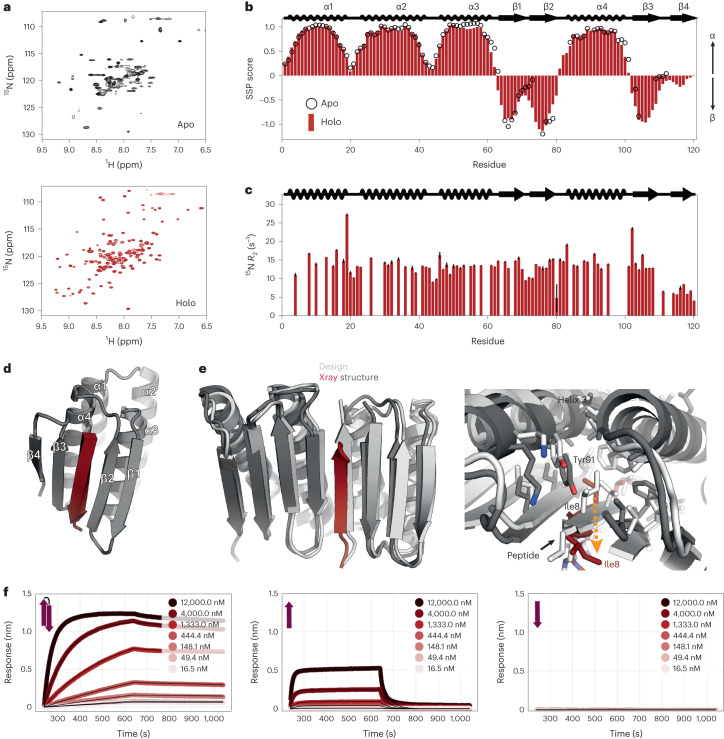
Structural characterization. **a**, NMR spectra of ^15^N-labeled C34 in the absence (top) and presence (bottom) of tenfold excess target peptide, 25 °C. **b**, Secondary structure propensity as a function of residue, based on backbone ^1^H, ^13^C and ^15^N chemical shifts recorded at 50 °C using the SSP program^34^. SSP scores for the apo form are shown with open circles, while those for the peptide-bound state are indicated with bars. The putative secondary structure of the designed protein is indicated above the plot. Positive values of SSP indicate α-helical structure, while negative values denote β-strands. **c**, ^15^N transverse relaxation rates as a function of residue. Low values, such as those in putative β4, indicate rapid timescale dynamics and are consistent with poorly formed structure. **d**, Designed model of C34. **e**, Left, overlay of the design model of a surface-redesigned version of C104 (gray) and the crystal structure (colors). Right; detailed interface view of the design (gray) and crystal structure (colors) with Ile8 shift indicated by the orange dashed arrow. **f**, Binding of CH15.1 to its hairpin peptide (left) or to the individual N-terminal strand (middle) or C-terminal strand (right) of the hairpin in BLI. Source data

**Extended Data Fig. 6:**
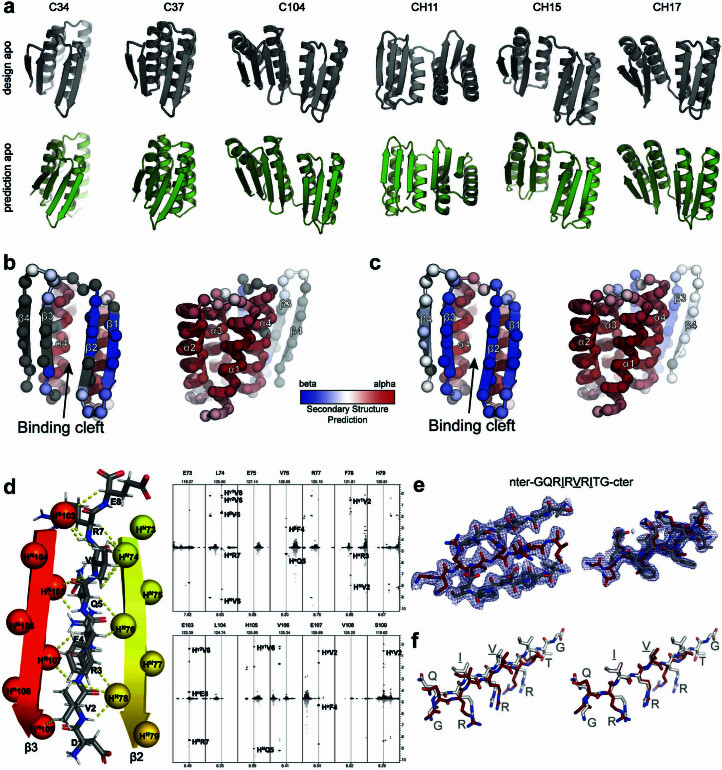
Structural characterization peptide binders. **a**, AlphaFold2 predictions of designed binder sequences in absence of peptide (bottom row) indicate closure of the binding pocket for some designs. **b**, Secondary structure prediction from NMR experiments on C34 apo mapped onto the cartoon model (two views) of C34 with C-alpha atoms shown as spheres (peptide not shown). No information available for residues in gray. These residues had broadened resonances due to conformational exchange. **c**, Same as **(b)** but for C34 holo (peptide not shown). **d**, Intermolecular NOE contacts between C34 and the peptide measured as previously described^58^ using a sample comprised of a mixture of 450 μM U-{^13^C,^15^N} C34 and 450 μM unlabeled peptide. Strips from the 3D dataset are illustrated at the ^15^N chemical shifts of the amides of the indicated residue (top of panels) showing the detected intermolecular contacts between the amide protons of strands β2/β3 from C34 and the peptide (right panel). The protons linked via the observed NOEs are highlighted on the structure of the designed binder on the left panel. **e**, Two atomic views with 2mF_obs_−DF_calc_ electron density maps contoured at 1.5σ of the strand-strand interaction between the binder and peptide of C104. **f**, Left, View of peptide in designed model after superposition of entire designed (white) and xray structures (red). Right superposition on only peptides in designed and xray structure.

In the absence of peptide, the binder contains a vacant cleft that exposes a hydrophobic core. Structure prediction methods predict that this cleft closes to form a continuous sheet in the apo state suggesting that the designs are structurally dynamic (Extended Data Fig. 6a). To study this we recorded a [^15^N,^1^H]-HSQC nuclear magnetic resonance (NMR) spectrum of unbound C34. The spectrum showed broadened resonances (Fig. 4a, top), suggesting the occurrence of exchange processes on the millisecond timescale. This prevents a straightforward structural characterization for most of the designed β-strand regions in the absence of the peptide; a large portion of putative strand β3 and the whole of putative strand β4 could not be assigned, and therefore the secondary structure propensities^34^ (open circles in Fig. 4b and Extended Data Fig. 6b) could not be calculated for residues within these regions. Further investigation^35^ of C34 showed that strands β3 and β4 are in equilibrium between two conformations with similar populations: one where the cleft closes by formation of parallel β2−β3 pairing (as in Extended Data Fig. 6a) and another where β4 replaces the peptide through formation of an antiparallel β1-β2-β4-β3 sheet. This contrasts with the structure predictions for apo C34, in which only the parallel β2−β3 pairing state is expected to form in the absence of peptide (Extended Data Fig. 6a).

In contrast to the free state, the NMR spectrum of the bound state shows sharp signals (Fig. 4a, bottom), indicating that the exchange process is quenched in the presence of the peptide. The secondary structure is as designed (Fig. 4b, red bars, and Extended Data Fig. 6c) except for β4, which has lower β-strand propensity as confirmed by ^15^N transverse relaxation (*R*_2_) experiments that indicate an increase in fast timescale dynamics in this region (Fig. 4c). We confirmed that the peptide binds in the designed orientation by measuring intermolecular nuclear Overhauser effect (NOE) contacts between it and C34 (Extended Data Fig. 6d).

We obtained a 2.3-Å-resolution crystal structure of a variant of C104, C104.1, where all the surface residues outside the interface were redesigned using ProteinMPNN^36^. The crystal structure recapitulates the designed model, with both individual domains clamping the peptide in a β-strand conformation (Fig. 4e and Supplementary Table 2). The individual domains superimpose well with the design model. The majority of the peptide is resolved in the electron density and binds in a β-strand conformation with the apolar residues buried in the designed cleft (Fig. 4e and Extended Data Fig. 6e). A deviation from the designed model at helix 3 shifts Tyr91 towards the peptide binding pocket in the crystal structure partially occluding it (Fig. 4e). As a result, peptide residue Ile8 is displaced (Extended Data Fig. 6f) and the last few residues of the peptide are disordered in the crystal and are not modeled (Methods).

While we were not able to obtain a crystal structure of a hairpin-binding design, strand deletion experiments support the idea that these peptides bind to the scaffold in a hairpin conformation rather than through single-strand insertion: the binding of individual strands of the CH15.1 hairpin to the CH15.1 binder is weaker than the binding of the whole hairpin by BLI (Fig. 4f).

### Design of amyloidogenic peptide-binding proteins

**Fig. 5:**
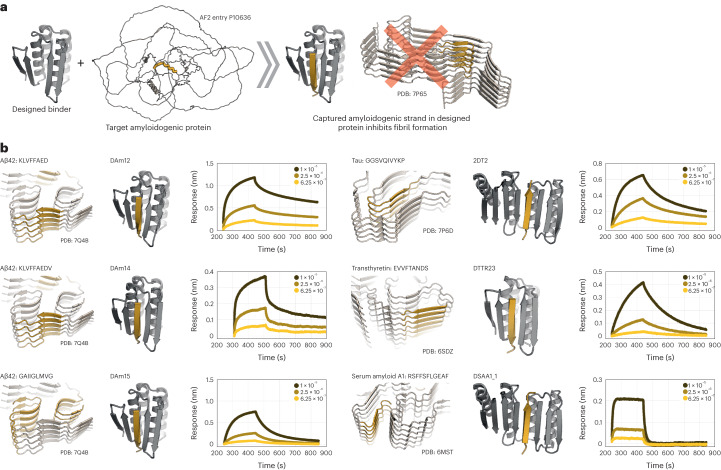
Design of amyloid peptide traps. **a**, Schematic illustration showing that designed amyloidogenic peptide binders (left, gray) can bind amyloidogenic sequences (yellow ribbon) that otherwise form amyloid fibrils through strand−strand interactions (right) and block fibril formation. AF2, AlphaFold2. **b**, Models of designs (middle column, designed binder in gray and target peptide in yellow) that bind amyloidogenic fragments (left column) from five different amyloid-forming proteins in BLI experiments (right column, legend in molar units). Source data

Encouraged by the biochemical and structural validation of our design approach on the two-sided binder design challenge, we next investigated whether the approach could generate binders to naturally occurring peptide or protein segments that form amyloids in a range of disease states. This is a more challenging ‘one-sided’ design problem because the target sequence is fixed. Amyloid fibril deposits can form both inside the central nervous system, as is the case for Aβ42, the microtubule-associated protein tau and α-synuclein, and also extracerebrally, as in the case of transthyretin- and serum amyloid A1-mediated amyloidosis^6,37,38^. The fibrils form through strand−strand-mediated oligomerization/fibrillization and are harmful to cells and tissues^8,9^. We aimed to design binders to fibril-forming regions—once bound to the designed scaffolds, these regions essentially become trapped and are likely unable to participate in fibril assembly (Fig. 5a).

To design such binders, we started from the design constraint that the peptide side chains facing the core of the binding scaffold must be primarily hydrophobic; because the peptide is bound in a β-strand conformation, every other residue is in the core and hence must be hydrophobic. We scanned the primary sequences of the Aβ42 peptide, microtubule-associated protein tau, transthyretin and serum amyloid A1 for regions that matched this pattern (Supplementary Fig. 4 and Methods). Matched regions were docked in a β-conformation into the binding cleft of the scaffolds, and the scaffold interface residues were redesigned to maximize contacts to the amyloid-derived β-strand, including surface-exposed hydrophobic interactions as described above. Designs with docked peptides predicted to participate in fibril or oligomer formation, based on experimentally determined amyloid structures^10–15^, were selected for experimental characterization.

**Extended Data Fig. 7:**
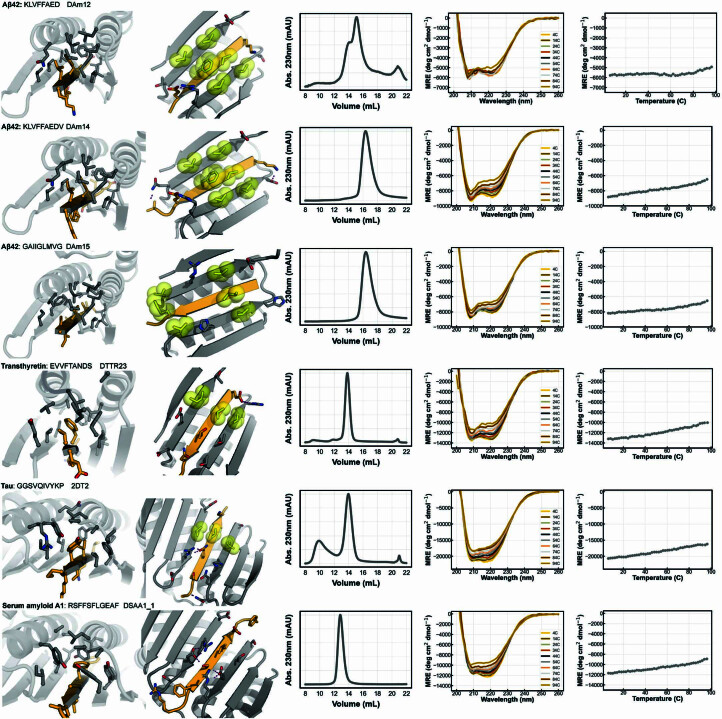
Characterization amyloidogenic strand binders. Close-up view of solvent inaccessible part interface (first column), close up view of solvent accessible part of interface with hydrophobic interaction pairs in yellow spheres and sticks (2nd column), SEC trace of purified binder on S75 increase 10/300GL (3rd column), CD wavelength scans (4th column) and CD temperature melt at 222 nm. Source data

The amyloid strand binders were first tested using the bicistronic expression screen described above; amyloid peptide fragments were fused to the C terminus of sfGFP and coexpressed with polyhistidine-tagged binder. Between 12 and 46 genes were tested depending on the target (Supplementary Table 4). After IMAC purification and SDS−PAGE we found that peptides derived from Aβ42, transthyretin, tau and serum amyloid A1 interacted with the binders. In roughly 25% of the cases, the binder and peptide fusion protein co-eluted in SEC, indicating that the complexes remained stably associated even when diluted on the column. The majority of the designed scaffolds were also stable and mostly monodisperse by SEC when purified in the absence of their target peptides (Extended Data Fig. 7 and Supplementary Table 4). We synthesized biotinylated versions of the single-strand Aβ42, transthyretin, tau and serum amyloid A1 fragments targeted by the designs and immobilized them on streptavidin biosensors for testing in BLI, identifying binders to all target peptides (Fig. 5b and Supplementary Table 4); we also observed some cross-reactivity consistent with similarities in the amyloid-forming sequences. For example, the Aβ42 binders DAm14 and DAm15 bound their target and also interacted with peptides derived from transthyretin and tau (Supplementary Fig. 5), but the binding signals were substantially lower in the off target cases. Circular dichroism spectroscopy and SEC experiments indicated that DAm14 and DAm15 were folded and thermostable, indicating that the promiscuous binding was not due to protein unfolding (Extended Data Fig. 7). Other designs such as for the transthyretin binder DTTR23, tau binder 2DT2 and serum amyloid A1 binder DSAA1_1 were more selective (Supplementary Figs. 5 and 6) toward their targets.

**Extended Data Fig. 8:**
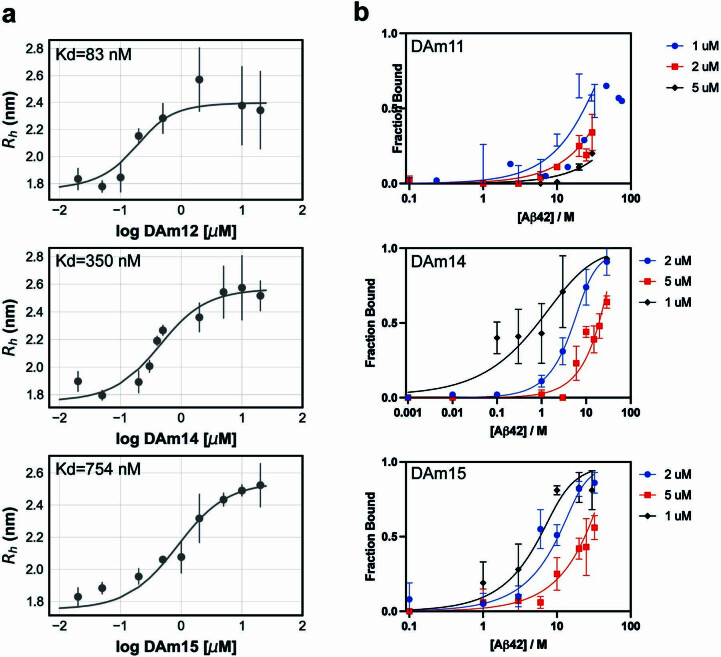
Microfluidic diffusional sizing binding experiments. **a**, Microfluidic diffusional sizing (MDS) binding isotherms of DAm12, DAm14 and DAm15 binding to Aβ42 monomers. Error bars represent SD (n = 3 independent measurements). **b**, MDS binding of pre-formed Aβ42 fibrils to designs DAm11 (top), DAm14 (middle) and DAm15 (bottom). Data for each individual measurement point are presented as mean +/− SD (n = 3) of independent replicates. Source data

We next investigated the binding properties of DAm12, DAm14 and DAm15 to the Aβ42 monomer using microfluidic diffusional sizing (MDS). Measurements indicated that DAm12 and DAm14 interacted with the monomeric form of the Aβ42 peptide with dissociation constants of 83 nM and 350 nM, whereas the *K*_d_ for DAm15 was 755 nM (Extended Data Fig. 8 and Supplementary Table 5). The designs also bound preformed Aβ42 fibrils (Extended Data Fig. 8).

### Designs potently inhibit Aβ42 fibril formation

**Fig. 6:**
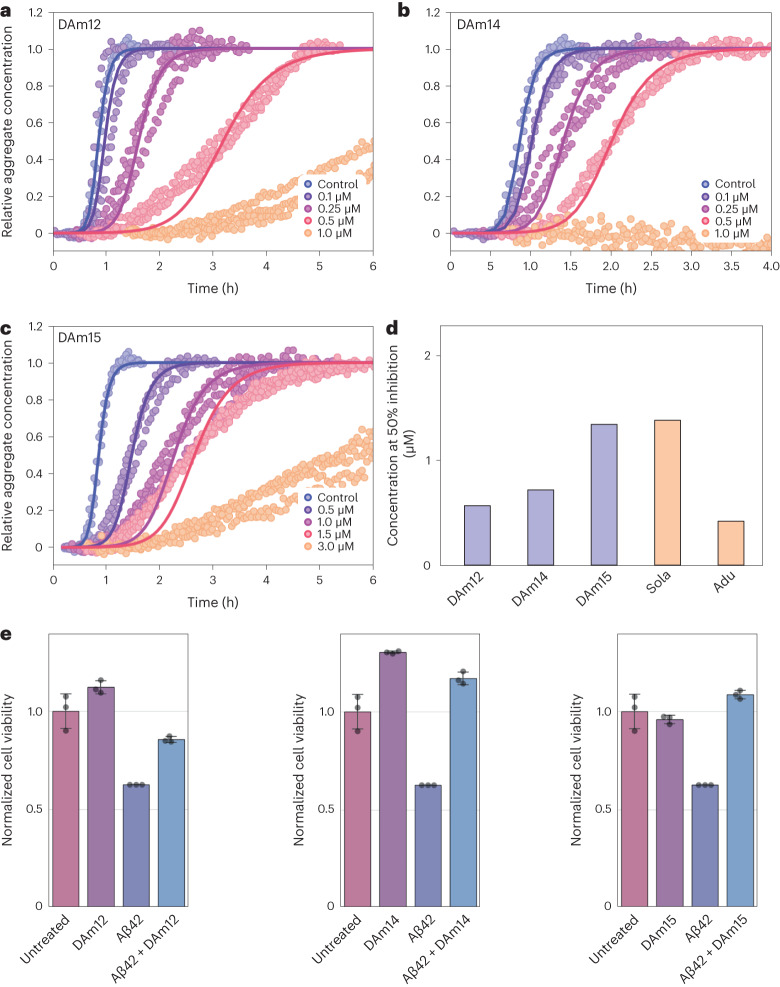
Inhibition of fibril formation. **a**−**c**, Aβ42 binders DAm12 (**a**), DAm14 (**b**) and DAm15 (**c**) strongly inhibit fibril formation at submicromolar concentrations in a ThT aggregation assay. Points are ThT fluorescence measurements; solid lines are fits of the kinetics expected when inhibitor binds Aβ42 monomer with the above-measured affinity and also inhibits secondary nucleation by direct interactions with the aggregates. **d**, Comparison of the Aβ42 aggregation inhibitory potential of the designed binders and clinical antibodies based on the concentration of inhibitor at which the aggregation reaction has been slowed by a fixed amount (that is, the half-time of aggregation (*t*_½_) is increased by 50%). Lower values indicate higher potency. The values for the clinical antibodies solanezumab (Sola) and aducanumab (Adu) are from ref. ^20^. **e**, DAm12, DAm14 and DAm15 protect neuroblastoma cells from Aβ42 toxicity. Cell viability was measured using the MTS assay at the aggregation half-time (where 50% of available Aβ42 protein has converted into aggregates and the highest concentration of cytotoxic oligomers is observed), in the presence of 1 µM Aβ42 and 2 µM of the designed binder. Data are presented as mean values ± s.d. (*n* = 3 individual wells as replicates). Source data

**Extended Data Fig. 9:**
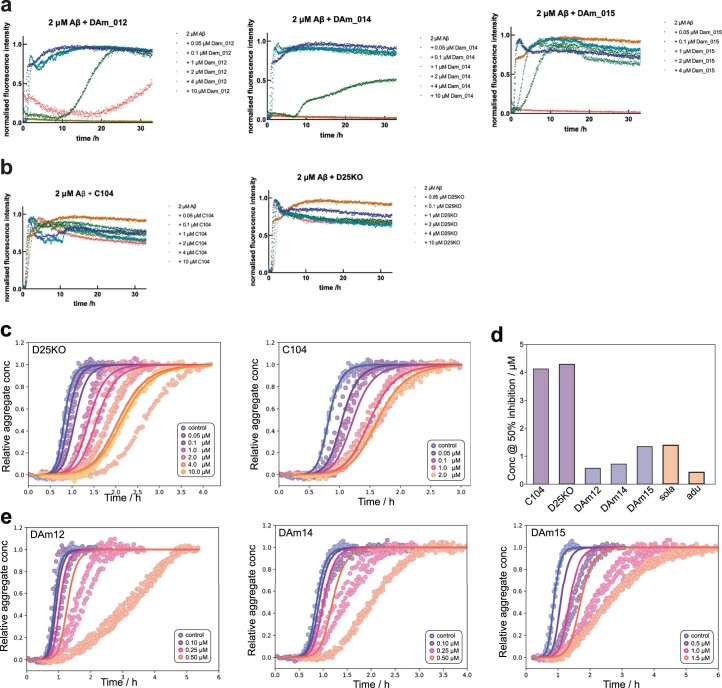
Aβ42 fibril inhibition. **a-c**, ThT Aβ42 fibril inhibition assays of the designed binders and controls that were not designed to inhibit Aβ42 aggregation. **d**, The inhibitory potential of binders, controls and clinical antibodies against Aβ42 aggregation is compared. See main Fig. 6d. **e**, Expected inhibitory effect due to monomer binding only. Points are ThT measurements, at a range of binder concentrations. The solid lines are produced by predicting the amount of inhibition at each binder concentration. To do so, we used the affinities of the binders to monomer to calculate the amounts of bound monomer and assumed that any monomer bound is completely removed from the aggregation reaction. Using the fits of the kinetics in the absence of binder, and the reaction orders determined previously^64^, we could then predict the expected inhibition at each binder concentration. Even for the tightest binders and assuming any bound monomer is permanently removed from the reaction, the observed inhibitory potential exceeds that expected to occur by monomer binding alone. This implies additional inhibitory mechanisms beyond interactions with monomeric Aβ42 are active. Source data

After characterizing the binding interaction between the binders and their targets, we next investigated the effect of the designs on amyloid fibril formation. To this end, we tested Aβ42 fibril formation in the presence of DAm12, DAm14 and DAm15 in a thioflavin T (ThT) assay. We observed rapid fibril formation in the control reactions (Fig. 6a–c and Extended Data Fig. 9), but in the presence of the binders, fibril formation was significantly retarded in a concentration-dependent manner, with DAm12 and DAm14 being more potent than DAm15, consistent with the tighter dissociation constants measured through MDS (Fig. 6a–c, Extended Data Fig. 8 and Supplementary Table 5). DAm14 and DAm15, at stoichiometric ratios, completely inhibited fibril growth for at least 30 h. DAm12 prevented detectable amyloid formation for 10 h even under a 1:2 substoichiometric ratio of inhibitor to peptide, comparable to clinical-stage therapeutic antibodies raised against this same target, including the approved drug aducanumab^20^ (Fig. 6d). Like DAm14 and DAm15, DAm12 is thermostable and remained folded at temperatures up to 94 °C in circular dichroism melting experiments (Extended Data Fig. 7). In a control experiment, C104 (Fig. 2a) and a previously de novo designed binder with a mixed α/β topology^27^ showed significantly lower inhibitory potential, indicating that the presence of a hydrophobic cleft surrounded by a β-sheet structure is insufficient for inhibition (Extended Data Fig. 9b–d).

To understand in more detail the mechanistic drivers of the observed inhibition, we used kinetic modeling^39^ to dissect the overall changes in the aggregation profiles in terms of changes in the underlying molecular rate constants. Chemical kinetic analysis showed that there is a contribution to the aggregation behavior from direct binding to the monomeric precursor peptide that is trapped by the designed binder, and an additional synergistic inhibitory effect from the interaction with fibrillar species (see fitted curves in Fig. 6, which include both effects). An inhibitory mechanism that includes interactions with aggregated species is supported by the observation that the binders interact directly with fibrils by MDS (Extended Data Fig. 8). Since the binders only interact with 8 or 9 of the 42 residues in Aβ42, binding to the fibrils could occur in regions where this part of the Aβ42 is not fully incorporated in the fibril, and suppress secondary nucleation (fitted curves in Fig. 6) as has been observed for the Brichos domain^40^.

To test the ability of the designs to protect cells from the toxic effects associated with protein aggregation, we challenged neuroblastoma cells with 1 µM of extracellular Aβ42 and followed cell viability using MTS (3-(4,5-dimethylthiazol-2-yl)-5-(3-carboxymethoxyphenyl)-2-(4-sulfophenyl)-2H-tetrazolium) reagent. Aggregates from the Aβ42 peptide decreased cell viability, an effect that has been shown to originate largely from oligomeric species (Fig. 6e)^40^. The addition of 2 µM of DAm12, DAm14 or DAm15 to the aggregating peptide solution before exposure to the cell culture medium fully protected the cells from toxic Aβ42 species (Fig. 6e). These results are consistent with our in vitro kinetic data (Fig. 6a–c) and indicate that the designs can protect cells from Aβ42-associated toxicity by preventing the formation of cytotoxic oligomers via trapping of Aβ42 monomers and inhibiting secondary nucleation.

## Discussion

We present a general approach for designing binders targeted to disordered stretches of proteins and peptides that can adopt β-strand or β-hairpin conformations. The designed binders are folded and bind the target peptides with nanomolar affinities in vitro and in cells and they can be incorporated into larger assemblies through fusion of the peptide or binder to other components. Binding hydrophobic regions of proteins is challenging because the properties that make proteins stick to hydrophobic surfaces can also lead to poor solubility and highly indiscriminate binding; the overall geometry of the designed binding pocket and the dynamic sheet opening/closure observed by NMR^35^ (Fig. 4) appear to limit such adverse effects. While the apo state is dynamic, the X-ray crystal structure of a designed binde−peptide complex is highly ordered and very close to the design model.

The shape-complementary binding pockets in our designs nearly completely engulf the bound peptide. This enables the capture of protein segments that are prone to amyloid formation such as those found in amyloid precursor protein, the microtubule-associated protein tau, transthyretin and serum amyloid A1. The designs potently inhibit the formation of the Aβ42 fibrils that are a hallmark of Alzheimer’s disease (AD), at a similar potency as clinically evaluated antibodies, including an approved drug (aducanumab). Based on kinetic modeling, we find that, in addition to monomer trapping, the designs may also act through blocking secondary nucleation sites. These designs provide valuable tools for testing hypotheses on, for instance, the role of small oligomeric toxic amyloid species (that consist of constituent monomers that should be captured by our designs), examining pathogenic cell-to-cell transmission of amyloidogenic proteins^41^ and monitoring of nonaggregated amyloidogenic species^42^.

After decades of failures in the clinic, there have recently been several very encouraging results in AD trials with antibodies targeting the Aβ42 peptide, leading to the approval of the first two disease-modifying drugs against AD (aducanumab and lecanemab), which exert their action through targeting the Aβ42 peptide and its aggregated forms. These clinical successes have led to a renewed interest in the Aβ42 peptide as the prime target in AD. Our designed Aβ binders have several potential advantages relative to the antibodies, including lower molecular weight, potential for increased stability and ability to modulate their binding properties. The fact that we have already achieved similar binding characteristics in vitro relative to clinical-stage antibodies is encouraging. An understanding of how and to what extent our Aβ42 inhibitors can be directly translated requires further study given the complexity of the disease and the requirement for blood−brain barrier (BBB) traversal. By contrast, for peripheral amyloidoses, where BBB traversal is not required, such as those caused by transthyretin or serum amyloid A1, a more direct route to testing of therapeutic efficacy of the designs could be available. Moving forward, the designs should also be useful as novel research tools for testing hypotheses on the biophysics of amyloid formation and the pathogenesis of AD and other amyloid diseases.

## Methods

### Protein design

#### Backbone generation

We explored two approaches to generate scaffolds with β-sheets with open slots for peptide β-strand insertion (Fig. 1 and Supplementary Fig. 1) using blueprint-based backbone building in Rosetta and PyRosetta4 (refs. ^43–47^). First, we explored using a two-domain binder approach (Supplementary Fig. 1a). We started from a scaffold, 2003285_0000, designed by FoldIt players^29^ (domain 1) and generated a β-sheet that extends from the C-terminal strand of the scaffold using blueprint-based backbone generation^43,45^. Then, this sheet was expanded into a second mixed α/β domain with three strands and one helix or four strands and two helices. The central strand of the β-sheet that encompasses both domains was split off from generating an individual peptide in β-strand conformation that can bind the designed deep cleft between domain 1 and domain 2. A connecting loop linking the helices that make up the interdomain interface was next generated using loop closure^48^ to yield a single-polypeptide two-domain binder that clamps the peptide on either side through β-strand backbone bonds (Supplementary Fig. 1a). The same approach was followed to generate β-hairpin-binding scaffolds.

In the second approach, a different FoldIt scaffold, 2003333_0006 (ref. ^29^), was modified to function as a peptide binder (Supplementary Fig. 1b). The connection between β-strands 3 and 4 was removed to create the individual peptide component. To stabilize the modified binder and ensure its solubility in the absence of peptide, we designed buttressing secondary structure elements that support the binding interface and scaffold. β-strand 3 was paired with another antiparallel strand, whereas helices 1 and 2 were backed up by either one or two supporting helices. After backbone generation, Rosetta combinatorial sequence design calculations were used to optimize the sequences of both the scaffold and the peptide for high-affinity binding. Designs with favorable interaction energy, few unsatisfied buried polar atoms and high shape complementarity, and for which Rosetta folding simulations yielded models close to the designed model, were selected for experimental characterization.

#### Sequence design

The amino acid sequences of the newly built polyvaline backbones were optimized using Rosetta flexible backbone enabled combinatorial side chain design followed by a second design round for the peptide-binder interface^32,49^. Ref2015, beta_nov16 or beta_genpot scorefunctions was used during design^50^. For a subset of designs, buried polar hydrogen-bond networks were designed using the HBNet mover^30^.

The affinity between peptide and binder was computationally improved by introducing hydrophobic interaction pairs to the solvent-exposed side of the interface. All solvent-exposed interaction pairs for which the Cα atoms were within 6 Å of each other were selected and allowed to be redesigned with the PackRotamersMover to only phenylalanine, alanine, methionine, isoleucine, leucine, tyrosine, valine and tryptophan using a fixed backbone. For computational affinity optimization of the natural target peptides, all surface exposed residues on only the binder within 6 Å of the target hydrophobic side chain were allowed to be redesigned. Residues around the redesigned interaction pairs were repacked. Single redesigned pairs and combinations of pairs were selected for experimental characterization.

To facilitate crystallization, the surface residues outside the interface were redesigned using ProteinMPNN^36^ for design C104. The structures of sequences obtained from ProteinMPNN were predicted using AlphaFold2 (ref. ^51^), and designs with rmsd ≤ 1.5 and plDDT ≥ 85 to the original designed model were selected for experimental characterization.

#### Design of rigid helical fusions

Rigid fusion of peptide binders and components of the LHD hetero-oligomer system was performed as described previously^28,52^.

#### Matching natural peptide sequences to scaffolds

The protein sequences of amyloid precursor protein, microtubule-associated protein tau, transthyretin and serum amyloid A1 were searched for burial patterns that were also present in the peptides of designs C34, C37, C104 and CH15. For C104, both the designed model and the crystal structure of C104, minimized with FastRelax^53^, were used. The burial patterns representing the relative positions of solvent-inaccessible residues versus solvent-accessible residues in the designed peptides were identified by visual inspection. For each peptide, all amyloidogenic protein sequence frames of length *n*, where *n* is the number of residues in the designed peptides mentioned above, were scanned for matching regions. Only residues phenylalanine, alanine, methionine, isoleucine, leucine, valine, serine, threonine, tyrosine or glycine residues were allowed at the solvent-inaccessible positions. At the remaining positions, all residues were allowed except for proline, which was only allowed at either terminus. The sequences against which matches were searched were DAEFRHDSGYEVHHQKLVFFAEDVGSNKGAIIGLMVGGVVIA for Aβ42, RSFFSFLGEAFDGARDMWRAYSDMREANYIGSDKYFHARGNYDAAKRGPGGVWAAEAISDARENIQRFFGHGAEDSLADQAANEWGRSGKDPNHFRPAGLPEKY for SAA1, PGGGKVQIINKKLDLSNVQSKCGSKDNIKHVPGGGSVQIVYKPVDLSKVTSKCGSLGNIHHKPGGGQVEVKSEKLDFKDRVQSKIGSLDNITHVPGGGNKKIETHKLTFRENAKAKTDHGAEIVYK for tau and GPTGTGESKCPLMVKVLDAVRGSPAINVAVHVFRKAADDTWEPFASGKTSESGELHGLTTEEEFVEGIYKVEIDTKSYWKALGISPFHEHAEVVFTANDSGPRRYTIAALLSPYSYSTTAVVTNPKE for transthyretin. For matching sequence stretches, we verified whether the matching sequence was also participating in β-strand interaction in amyloid fibrils based on published cryo-electron microscopy structures of amyloid fibrils (see main text for references). When this was the case, the sequence of the template designed peptide was mutated to the sequence of the matched sequence of the amyloidogenic protein. The resulting peptide−binder complex was minimized, and the residues in the interface of the designed binder were redesigned to optimally match the amyloidogenic sequence by also including hydrophobic interaction pairs across the solvent-accessible area of the interface (see above). The Protein Data Bank (PDB) models of the designed proteins and example scripts can be downloaded as source data.

#### Protein expression and purification

Synthetic genes encoding designed proteins were purchased from Genscript or Integrated DNA Technologies (IDT) in the pET29b expression vector or as eBlocks (IDT) and cloned into customized expression vectors^54^ using GoldenGate cloning. A His6x tag was included either at the N terminus or at the C terminus as part of the expression vector. In some cases, a tobacco etch virus (TEV) protease recognition site was introduced at the N terminus after the histidine tag. Peptide genes were purchased as fusion proteins to either the C terminus of sfGFP or the N terminus of a ubiquitin−AviTag−His6x construct separated by a Pro-Ala-Ser linker. Bicistronic genes were ordered as described^28^. Detailed construct information is provided in the Supplementary Data 1.

Proteins were expressed using autoinducing medium consisting of TBII medium (Mpbio) supplemented with 50× 5052, 20 mM MgSO_4_ and trace metal mix in BL21 LEMO *E.* *coli* cells. Proteins were expressed under antibiotic selection at 37 °C overnight or at 18–25 °C overnight after initial growth for 6–8 h at 37 °C. Cells were harvested by centrifugation at 4,000*g* and resuspended in lysis buffer (100 mM Tris pH 8.0, 200 mM NaCl, 50 mM imidazole pH 8.0) containing protease inhibitors (Thermo Scientific) and bovine pancreas DNase I (Sigma-Aldrich) before lysis by sonication. The reducing agent TCEP (1 mM final concentration) was included in the lysis buffer for designs with free cysteines. Proteins were purified by IMAC. Cleared lysates were incubated with 2–4 ml nickel NTA beads (Qiagen) for 20–40 min before washing the beads with 5–10 column volumes of lysis buffer, 5–10 column volumes of high-salt buffer (10 mM Tris pH 8.0, 1 M NaCl) and 5–10 column volumes of lysis buffer. Proteins were eluted with 10 ml of elution buffer (20 mM Tris pH 8.0, 100 mM NaCl, 500 mM imidazole pH 8.0). His6x tags were cleaved by dialyzing IMAC elutions against 20 mM Tris pH 8.0, 100 mM NaCl, 1 mM TCEP overnight in the presence of His6x-tagged TEV protease followed by a second IMAC column to remove His6x−TEV and uncleaved protein.

Single-cysteine variants of DAm12, DAm14 and DAm15 were purified as described above and labeled with Alexa488-C5-maleimide (Thermo) at a concentration of between 50 and 100 μM of protein and a twofold to fivefold molar excess of label in SEC buffer supplemented with 1 mM TCEP protected from light. After 3 h at room temperature or overnight at 4 °C, the labeling reaction was quenched by the addition of 1 M dithiothreitol (DTT).

As a final step, all protein preparations were polished using SEC on either Superdex 200 Increase 10/300GL or Superdex 75 Increase 10/300GL columns (Cytiva) using 20 mM Tris pH 8.0, 100 mM NaCl. The reducing agent TCEP was included (1 mM final concentration) for designs with free cysteines. For designs where a substantial void volume peak was present in addition to the monomer peak, the monomer peak was pooled and reinjected. Only designs where, upon reinjection, the void peak was mostly absent were further pursued. SDS−PAGE and LC−MS were used to verify peak fractions. Proteins were concentrated to concentrations between 0.5–10 mg ml^−1^ and stored at room temperature or flash frozen in liquid nitrogen for storage at −80 °C. Thawing of flash-frozen aliquots was done at room temperature or 37 °C. All purification steps from IMAC were performed at ambient room temperature.

The C104.1 complex was prepared by incubating the binder with a threefold to fivefold molar excess of the peptide for 3 h at room temperature followed by SEC.

#### Peptide synthesis

All Fmoc-protected amino acids were purchased from P3 Bio. The biotinylated peptides obtained by synthesis were padded at the C terminus with SGGSGG-Kbiotin, where Kbiotin is a Fmoc-Lys(biotin)-OH building block also purchased from P3 Bio. Oxyma was purchased from CEM, and DIC was purchased from Oakwood Chemicals. Dimethylformamide was purchased from Fisher Scientific and treated with an AldraAmine trapping pack (Sigma-Aldrich) before use. Piperidine was purchased from Sigma-Aldrich. Cl-TCP(Cl) resins were purchased from CEM. The peptides were synthesized on a 0.1 mmol scale using microwave-assisted solid-phase peptide synthesis via a CEM Liberty Blue system and subsequently cleaved with a cleavage cocktail consisting of trifluoroacetic acid (TFA), TIPS, water and DODT (92.5:2.5:2.5:2.5 in order). The cleavage solution was concentrated in vacuo, precipitated into cold ether and spun down by centrifugation. The pellet was washed and spun down again with ether (2⨯) and then dried under nitrogen, resuspended in water and aceyonitrile (ACN) and purified by RP-HPLC on an Agilent 1260 Infinity semi-prep system with a gradient from 20% to 70% over 15 min (A: H_2_O with 0.1% TFA; B: ACN with 0.1% TFA). The purified peptide fractions were combined into one, lyophilized and massed in a tared scintillation vial for the final product. Peptides derived from transthyretin, tau and serum amyloid A1 were purchased from WuXi. Depending on the isoelectric point, lyophilized peptides were solubilized in buffers containing either 100 mM Tris pH 8.0 or 100 mM MES pH 6.5 and stored at −20 °C.

#### Mammalian cell culture and transfection

HeLa cells (ATCC, CCL-2) were cultured in DMEM (Gibco) supplemented with 1 mM l-glutamine (Gibco), 4.5 g l^−1^ d-glucose (Gibco), 10% FBS and (1×) nonessential amino acids (Gibco). Cells were kept in culture at 37 °C and 5% CO_2_ and split twice per week by trypsinization using 0.05% trypsin EDTA (Gibco) followed by passage at 1:5 or 1:10 into a new tissue culture-treated T75 flask (Thermo Scientific, 156499). Before transfection, cells were plated at 20,000 cells per well on CELLview cell culture slides (Greiner Bio-One, 543079) for 24 h, after which transfection took place using 187.5 ng of total DNA per well and 1 μg μl^−1^ PEI-MAX (Polyscience) mixed with Opti-MEM medium (Gibco). Transfected cells were incubated at 37 °C and 5% CO_2_ for 24 to 36 h before being imaged.

#### Fluorescence microscopy

Three-dimensional images were acquired with a commercial OMX-SR system (GE Healthcare) using a 488-nm Toptica diode laser for excitation. Emission was collected on a PCO.edge sCMOS camera using an Olympus ×60 1.42-NA PlanApochromat oil immersion lens. Images of 1,024 × 1,024 (pixel size, 6.5 μm) were captured without binning. AcquireSR acquisition control software was used for data collection. *z* stacks were collected with a step size of 500 nm and 15 slices per image. The images were deconvolved with an enhanced ratio using SoftWoRx 7.0.0 (GE Healthcare). Finally, cell images were sum-projected using ImageJ2 v2.1.0. and v2.3.0. Scale bars equal 10 µm.

#### Biolayer interferometry

BLI experiments were performed on an OctetRED96 BLI system (ForteBio) at room temperature in Octet buffer (10 mM HEPES pH 7.4, 150 mM NaCl, 3 mM EDTA, 0.05% surfactant P20) supplemented with 1 mg ml^−1^ BSA (Sigma-Aldrich). Before taking measurements, streptavidin-coated biosensors were first equilibrated for at least 10 min in Octet buffer. Chemically synthesized peptides with C-terminal biotin or enzymatically biotinylated peptide fusion proteins (see Supplementary Tables 6 and 7 for details) were immobilized on the biosensors by dipping them into a solution with 100−500 nM protein until the response reached between 10% and 50% of the maximum value followed by dipping the sensors into fresh Octet buffer to establish a baseline for 60 s. Titration experiments were performed at 25 °C while rotating at 1,000*g*. Association with designs was allowed by dipping the biosensors in solutions containing designed protein diluted in Octet buffer until equilibrium was approached, followed by dissociation by dipping the biosensors into fresh solution and monitoring the dissociation kinetics. In the peptide-binding cross-specificity assays, each biotinylated peptide was loaded onto streptavidin biosensors in equal amounts followed by 2 min of baseline equilibration. Then, association and dissociation with all the different binders was allowed for 400 s for each step. For the designed peptide−binder pairs, binder concentrations were around the *K*_d_ of the interaction between the loaded peptide and its designed binding partner, whereas the concentrations for the amyloid binders were 10, 2.5 and 0.625 μM. Global kinetic or steady-state fits were performed on buffer-subtracted data using the manufacturer’s software (Data Analysis 9.1) assuming a 1:1 binding model. Data acquisition was performed using OctetRed96 data acquisition software 9.

#### Enzymatic biotinylation of proteins

Proteins with Avi tags (GLNDIFEAQKIEWHE; Supplementary Tables 6 and 7) were purified as described above and biotinylated in vitro using the BirA500 (Avidity) biotinylation kit. Protein (840 μl) from an IMAC elution was biotinylated in a 1,200-μl (final volume) reaction according to the manufacturer’s instructions. Biotinylation reactions were allowed to proceed at either 4 °C overnight or for 2−3 h at room temperature on a rotating platform. Biotinylated proteins were purified using SEC on a Superdex 200 column (Increase 10/300 GL, GE Healthcare) or an S75 Increase 10/300 GL column (GE Healthcare) using SEC buffer (20 mM Tris pH 8.0, 100 mM NaCl).

#### Circular dichroism spectroscopy

Circular dichroism spectra were recorded in a cuvette with a 1-mm path length at a protein concentration between 0.3–0.5 mg ml^−1^ on a J-1500 instrument (Jasco). For temperature melts, data were recorded at 222 nm every 2 °C between 4 and 94 °C and wavelength scans were done between 190 and 260 nm at 10 °C intervals starting from 4 °C. Experiments were performed in 20 mM Tris pH8.0, 20 mM NaCl. The high-tension voltage was monitored according to the manufacturer’s recommendation to ensure optimal signal-to-noise ratio for the wavelengths of interest.

#### SEC binding assays

SEC binding assays between purified designs and GFP−peptide fusions were performed on a Superdex 75 Increase 10/300 GL column (Cytiva) in 20 mM Tris pH 8.0, 100 mM NaCl using 500-μl injections containing a 15 or 20 μM final concentration of each component. Binding reactions were allowed to equilibrate for at least 45 min before injection. For the subunit exchange experiment, the disulfide-stabilized complex between C104.2 and ubiquitin−pep104.2 as well as the control base noncovalent complex was allowed to form overnight at a 20 μM equimolar concentration under oxidizing conditions, after which competing GFP−pep104 was added to the preformed complexes to a final concentration of 20 μM. After at least 45 min, the reaction was injected onto an SEC column. Elution profiles were collected by monitoring absorbance at 230 nm and 395 nm (absorbance of GFP). All experiments were performed at room temperature. Data were analyzed and acquired using the manufacturer’s Unicorn 7.3 software.

#### Disulfide formation assay

Individual protein components were purified as described above in the presence of 1 mM TCEP except for in the last SEC step, where no reducing agent was present. Reactions were incubated at room temperature using 50 μM of each component in 20 mM Tris pH 8.0, 100 mM NaCl. Reactions were stopped by adding an equal volume of 2⨯ nonreducing SDS protein-loading buffer at the indicated time points.

#### NMR

All NMR experiments for C34 were performed on Bruker Avance III HD 14.1 T or 18.8 T spectrometers equipped with cryogenically cooled x,y,z pulse-field gradient triple resonance probes. Resonance assignments were obtained by triple resonance (HB)CBCA(CO)NNH, HNCACB, HNCO, HN(CA)CO and HNN experiments^55^ acquired using U-^13^C,^15^N-labeled samples. Note that the spectra shown in Fig. 3a were recorded at 25 °C, but the resonance assignment for free C34 was done at 50 °C to reduce the line broadening arising from conformational exchange. Information from ^13^Cα, ^13^Cβ, ^13^CO, ^15^N and ^1^HN chemical shifts was combined into a single secondary structure propensity (SSP) score representing the expected fraction of α-structure or β-structure ^15^N *R*_2_ rates for the bound state of C34 were measured using the in-phase Carr−Purcell−Meiboom−Gill (CPMG) experiment^56^ with *ν*_CPMG_ = 1 kHz, *T*_*r*elax_ = 30 ms and CPMG refocusing pulses applied at a γB_1_/2π = 5.7 kHz field and phase-modulated according to the (*x*,*x*,*y*,-*y*) cycling scheme^57^. A NOESY dataset for recording intermolecular NOEs was acquired as previously described^58^ with a mixing time of 150 ms, using 450 μM of U-^13^C,^15^N-labeled C34 and 450 μM of unlabeled peptide at natural isotopic abundance. Data were acquired using topspin3.2 and topspin3.5. Data were analyzed using nmrPipe 11.0 and CARA 1.9.1.7.

#### Aβ expression, purification and labeling

Aβ42 peptide was expressed and purified as reported previously^59^. In short, the synthetic gene encoding NT*FlSp was purchased from Genscript (Genscript Biotech), ligated into pT7 plasmid containing a TEV recognition site (TRS) for Aβ42 (ref. ^60^) and transformed into chemically competent *E. coli* BL21 (DE3) cells and expressed as described previously^61^. Upon cleavage of the fusion protein with TEV protease, the sample was dissolved in 15 ml of 8 M guanidine-hydrochloride (GuHCl) and monomeric Aβ was purified on a Superdex 26/600 30-pg size exclusion column and lyophilized as aliquots until further use. To generate fibrils, several aliquots of lyophilized Aβ were combined for an increased protein concentration by dissolving aliquots in 1 ml of 8 M GuHCl and subjecting them to SEC on a Superdex 75 10/300 Increase column in 20 mM sodium phosphate, 0.2 mM EDTA buffer at pH 8.0. Subsequently, collected monomeric peptide, typically at a concentration of 30 µM, was pipetted into PEGylated plates (Corning, 3881) and incubated at 37 °C in a plate reader, with 100 rpm orbital shaking. To track the degree of monomer conversion into fibrils, ThT was added exclusively to control wells, and after the plateau was reached, the fibrils were harvested from ThT-free sample wells. To perform binding experiments of monomeric Aβ with the binders, a cysteine-carrying Aβ mutant (S8C) was expressed and purified as described previously^62^. Briefly, the plasmid carrying synthetic genes with *E. coli* optimized codons for the S8C mutant (developed by Thacker and colleagues and purchased from Genscript) were transformed into the BL21 DE3 pLysS star *E. coli* strain and the protein was expressed in auto-induction medium^63^. Upon purification using IEX and subsequent SEC on a 26 × 600 mm Superdex 75 column, the S8C monomer was eluted in sodium phosphate buffer supplemented with 3 mM DTT to prevent its dimerization and then lyophilized. For conjugation of the protein with a fluorescent dye, the lyophilized fractions were dissolved in 8 M GdnHCl and subjected to SEC in buffer without DTT before adding Alexa Fluor 488 dye (ThermoFisher) in at least 5× molar excess. The protein−dye mixture was incubated overnight at 4 °C, the free dye was removed via column chromatography and the protein was used immediately.

#### Kinetic assays of fibril inhibition

Aliquots of purified lyophilized Aβ were dissolved in 8 M GuHCl and the monomeric protein was isolated by gel filtration on a Superdex 75 10/300 Increase column in 20 mM sodium phosphate, 0.2 mM EDTA buffer at pH 8.0. Samples were prepared on ice, using careful pipetting to avoid the introduction of air bubbles, and pipetted into a 96-well half-area plate of PEGylated black polystyrene with a clear bottom (Corning 3881), 100 μl per well, with three to four replicates per sample. All samples diluted with buffer to the final concentration of 2 μM Aβ were supplemented with 6 μM ThT (Sigma), with a range of concentrations of the binders per experiment. The kinetic assays were initiated by placing the 96-well plate at 37 °C under quiescent conditions in a plate reader (FLUOstar Optima, BMGLabtech). The ThT fluorescence was measured through the bottom of the plate every 165 s with a 440-nm excitation filter and a 480-nm emission filter.

#### Analysis of aggregation kinetics

Integrated rate laws describing the aggregation of Aβ42 were derived previously^64^. They reproduce well the kinetic curves obtained in ThT assays and can be used to quantify inhibitory effects. Here, we used the amylofit platform^39^ to determine the rate constants of aggregation in the absence of an inhibitor. Using the affinities of binder to monomer determined by MDS, we then calculated the concentrations of monomer expected to be bound at each binder concentration. Assuming all monomer bound is completely removed from the aggregation reaction (that is, ignoring dissociation of the monomer−binder complex over the timescale of aggregation), the effect of binders on the aggregation reaction is the same as a lowering of the monomer concentration. The kinetic curves resulting from this effective reduction of the monomer concentration were then computed using the amylofit platform (Extended Data Fig. 9), and the effect was found to be insufficient to explain the observed degree of inhibition. We then explored whether the presence of an additional mechanism of inhibition, by interaction with aggregated species, was able to describe the observed aggregation. To model this additional inhibition, we allowed the rate of secondary nucleation to vary with binder concentration, as detailed previously^39^. These results are shown as solid lines in Fig. 6 and effectively describe the inhibition at substoichiometric binder concentrations. At higher binder concentrations, when the majority of monomer is expected to be bound, these fits perform less well and thus only the experimental measurements, not the fits, are shown at the highest binder concentrations.

#### Cell viability assay

Cell viability assays were performed on SHSY-5Y human neuroblastoma cells cultured under standard conditions at 37 °C in a humidified incubator with 5% CO_2_. Cells were seeded at a density of 25,000 cells per well in a white-walled, clear-bottomed 96-well plate and cultured for 24 h in DMEM supplemented with 10% FBS. The culture medium was then replaced with phenol red-free DMEM without serum, supplemented with an antibiotic-antimycotic agent. Aβ monomer was isolated by gel filtration in 20 mM sodium phosphate buffer at pH 8.0 (without EDTA), mixed with the binders at a ratio of 10 µM:20 µM Aβ to binder, and stored on ice until further use. Samples used in the treatment of the cells were prepared by incubation in a 96-well nonbinding plate (Corning, 3881) at 37 °C so that the progress of aggregation could be tracked by ThT fluorescence in the control wells. Aliquots of corresponding ThT-free samples were taken when the reaction reached *t*_½_ (when 50% of full aggregation was reached, corresponding to the highest concentration of cytotoxic oligomeric species^64,65^) and immediately diluted tenfold in medium and applied to cells. The cells were then cultured in the presence of the peptides or buffer for an additional 24 h before the viability assays were performed. Cell viability was measured with CellTiter 96 AQ_ueous_ One MTS reagent from Promega. The MTS reagent was added to the cell culture medium and incubated with the cells at 37 °C in a humidified incubator with 5% CO_2_ for 1 h before the absorbance at 495 nm was measured in an Optima FLUOstar plate reader. All values given for the assay account for the positive control (2% Triton X-100) values as a baseline readout and are normalized relative to the untreated cells.

#### Microfluidic diffusional sizing

The binding affinity of the binders and monomeric Aβ was measured on a Fluidity One-M instrument (Fluidic Analytics). Fluorescently labeled Aβ mutant was mixed with unlabeled binders at a range of concentrations and incubated on ice for at least 30 min. Before the measurements, microfluidic circuits of the Fluidity One-M chip plate were primed using sample buffer. To create a binding curve for individual designs, each one of the different Aβ−binder mixtures was measured in triplicate. *K*_d_ values were determined by nonlinear least squares fitting as described previously^66^ using Prism (GraphPad Software). For MDS experiments concerning interactions of binders with Aβ fibrils, microfluidic devices were fabricated and operated as described previously^67,68^. In brief, the microfluidic devices were fabricated in PDMS using standard soft-lithography techniques and bonded onto a glass coverslip after activation with oxygen plasma. Sample loading from reservoirs connected to the respective inlets and control of flow rate were achieved by applying negative pressure at the outlet using a glass syringe (Hamilton) and a syringe pump (neMESYS, Cetoni). Images were recorded using a custom-built inverted epifluorescence microscope fitted with a fluorescence filter set with an excitation filter at 475 ± 35 nm, emission filter at 525 ± 30 nm and dichroic mirror for 506 nm (Laser, 2000) for detection of Alexa 488-labeled binders. Images were acquired using Micro Manager, typically at flow rates of 60 and 100 μl h^−1^, and lateral diffusion profiles were recorded at four different positions along the microfluidic channels. Diffusion profiles extracted from fluorescence images and confocal recordings were fitted using a custom-written analysis software by numerical model simulations solving the diffusion–advection equations for mass transport under flow^69^.

#### Crystal structure determination

The C104.1 complex (19 mg ml^−1^) was crystallized using the vapor diffusion method at room temperature in 0.1 M Tris pH 7.8, poly(-γ-glutamic acid) low-molecular-weight polymer, 15% PEG 4000 (Molecular Dimensions) and the crystals were harvested in 25% glycerol as a cryoprotectant. Data were collected from a single crystal at 100 K and 0.97918 Å at the Advanced Photon Source at Argonne National Laboratory. Diffraction images were integrated using XDS^70^ or HKL3000 (ref. ^71^) and merged/scaled using the AIMLESS application from the CCP4-7.0.076 software suite^72^. Starting phases were obtained by molecular replacement using Phaser^73^ from within CCP4-7.0.076, using the computational design models of the individual N-terminal and C-terminal domains of C104.1 as search models. Structures were refined using either phenix.refine^74^ or Refmac^75^ and PDB-REDO^76^. Model building was performed using COOT^77^. The lack of density at the C terminus of the peptide prompted us to examine the possibility of a β-strand register shift for peptide binding. OMIT maps were used to decrease the model bias. In addition, the peptide was modeled in several off-target β-strand registers. Overall, refinement statistics and *B* factors were better for the model where the peptide was modeled in the designed on-target β-strand register. The final model had 96.8% of residues in the favorable region of the Ramachandran plot and no outliers. The model was evaluated using MolProbity^78^. Data collection and refinement statistics are recorded in Supplementary Table 3. Data deposition, atomic coordinates and structure factors reported in this paper have been deposited in the PDB (http://www.rcsb.org/) with accession code 8FG6.

#### Statistics and reproducibility

Unless stated otherwise, all experimental results were reproduced at least two times with two different preparations of protein reagents. Many of the BLI binding experiments were performed three or more times with three to five protein preparations that were purified independently.

### Reporting summary

Further information on research design is available in the Nature Portfolio Reporting Summary linked to this article.

## Online content

Any methods, additional references, Nature Portfolio reporting summaries, source data, extended data, supplementary information, acknowledgements, peer review information; details of author contributions and competing interests; and statements of data and code availability are available at 10.1038/s41589-024-01578-5.

### Supplementary information

Supplementary Information Supplementary Tables 1–5 and Figs. 1–6.

Reporting Summary

Supplementary Table 6 Amino acid sequences of designs.

Supplementary Table 7 Mammalian cell constructs.

Supplementary Data 1 Source data for supplementary figures.

### Source data

Source Data Fig. 2 Statistical source data.

Source Data Fig. 3 Uncropped image.

Source Data Fig. 4 Statistical source data.

Source Data Fig. 5 Statistical source data.

Source Data Fig. 6 Statistical source data.

Source Data Extended Data Fig. 1 Statistical source data.

Source Data Extended Data Fig. 2 Statistical source data.

Source Data Extended Data Fig. 3 Statistical source data.

Source Data Extended Data Fig. 4 Statistical source data.

Source Data Extended Data Fig. 5 Statistical source data.

Source Data Extended Data Fig. 7 Statistical source data.

Source Data Extended Data Fig. 8 Statistical source data.

Source Data Extended Data Fig. 9 Statistical source data.

## Data Availability

Data supporting the main findings of the study are provided within the article and its Supplementary Information. Data that were too large to contain within the manuscript can be accessed in the open-access repository Zenodo^79^ (10.5281/zenodo.10391229). The crystal structure is available in the Protein Data Bank (8FG6). Structural models of designed proteins can be found as source data. Source data are provided with this paper.

## Code availability

Computer code and scripts can be downloaded from the open-access repository Zenodo (10.5281/zenodo.10391229).
