# Supplementary material for: Design of amyloidogenic peptide traps
Source: Nat Chem Biol. 2024 Mar 19;20(8):981–90. doi: 10.1038/s41589-024-01578-5 (PMC11288891; doi:10.1038/s41589-024-01578-5)
Supplement: Supplementary file 1 — Supplementary Tables 1–5 and Figs. 1–6. [file 41589_2024_1578_MOESM1_ESM.pdf]

nature chemical biology

Article

https://doi.org/10.1038/s41589-024-01578-5

Design of amyloidogenic peptide traps

In the format provided by the
authors and unedited

Supplementary Table 1. Overview designs

Design	Target	Target peptide sequence	Designed pocket type
**C34**	designed peptide	DVRFQVRE	single strand
**C34.1**	designed peptide	DVTFIVHE	single strand
**C37**	designed peptide	DVRFNFRE	single strand
**C104**	designed peptide	GQRIRVRITG	single strand
**C104.1**	designed peptide	GQRIRVRITG	single strand
**C104.2**	designed peptide	GQRICVRITG	single strand
**C104.3**	designed peptide	GQRIRVCITG	single strand
**CH11**	designed peptide	SQTHFEVEFKGMRIRLRNS	hairpin
**CH15**	designed peptide	SQTQFEYEKNGRRIRLRQS	hairpin
**CH15.1**	designed peptide	GWLEFEYEKNGRVIRLVQG	hairpin
**CH17**	designed peptide	SSVRVEEHMNGVRIQMEYG	hairpin
**DAm12**	Aβ42	KLVFFAED	single strand
**DAm14**	Aβ42	KLVFFAEDV	single strand
**DAm15**	Aβ42	GAIIGLMVG	single strand
**DTTR23**	Transthyretin	EVVFTANDS	single strand
**2DT2**	Tau	GGSVQIVYKP	single strand
**DSAA1_1**	SAA1	RSFFSFLGEAF	single strand

Supplementary Table 2. Biolayer interferometry global kinetic fitting parameters.

	K_D (µM)	k_{on} (M^{-1} s^{-1})	k_{off} (s^{-1})	chi-sqr	R-sqr
**C34**	12.8 ± 0.38	$1.37 \cdot 10^4$ ± 391	0.176 ± 0.0016	0.15	0.99
**C37**	0.31 ± $6.9 \cdot 10^{-4}$	547.4 ± 1.1	$1.7 \cdot 10^{-4}$ ± $1.8 \cdot 10^{-7}$	8.7	0.99
**C104**	0.157 ± 0.0015	6348 ± 56	$9.96 \cdot 10^{-4}$ ± $3.9 \cdot 10^{-6}$	0.48	0.99
**CH11**	0.167 ± $2.78 \cdot 10^{-3}$	4990 ± 59.1	$8.3 \cdot 10^{-4}$ ± $9.7 \cdot 10^{-6}$	0.11	0.99
**CH15**	44 ± 11.2	434 ± 10.9	0.019 ± $9.9 \cdot 10^{-5}$	0.02	0.99
**CH17**	9.4 ± 0.31	1155 ± 39	0.0146 ± $3.11 \cdot 10^{-4}$	0.064	0.99
**C34.1**	2.3 ± 0.017	1358 ± 9.9	$3.08 \cdot 10^{-3}$ ± $6.38 \cdot 10^{-6}$	7.34	0.99
**CH15.1**	0.05 ± $4.1 \cdot 10^{-4}$	6730 ± 34.4	$3.3 \cdot 10^{-4}$ ± $2.2 \cdot 10^{-6}$	0.46	0.99

Supplementary Table 3. Data collection and refinement statistics (molecular replacement)

	**C104.1 (PDB code:** 8FG6**)**
**Data Collection**	
Space group	P 31 2 1
Cell dimensions	
a, *b*, *c* (Å)	64.54, 64.54, 112.61
α, *β*, *γ* (°)	90, 90, 120
Resolution (Å)	55.89 - 2.30 (2.53 - 2.30)*
R_{merge}	0.118 (0.966)
$I/\sigma(I)$	12.97 (2.87)
Completeness (%)	99.50 (99.51)
Redundancy	13.6 (14.3)
**Refinement**	
Resolution (Å)	55.89 - 2.30 (2.53 - 2.30)
No. reflections	12514 (3052)
R_{work} / R_{free}	0.2552 (0.3551)/ 0.2917 (0.3573)
No. atoms	
Protein	1288
Water	13
B-factors	
Protein	50.91
Water	53.79
R.m.s. deviations	
Bond lengths (Å)	0.013
Bond angles (°)	1.65

*Values in parentheses are for highest-resolution shell.

Supplementary Table 4. Success rates amyloid binder design.

Target	Ordered bicistronic designs	Complex formation in bicistronic screen	Monodisperse monomer	Robust binding signal BLI	Overall success rate
Aβ42	35	7	4	3	9%
Tau	12	4	4	1	8%
Transthyretin	46	10	6	1	2%
SAA1	30	9	5	1	3%

Supplementary Table 5. Binding constants microfluidic diffusional sizing

Design	K_D (nM)	95%CI
DAm11	3290	1240 - 8240
DAm12	83	15.72 - 217.3
DAm14	350	183.4 - 635.4
DAm15	754	378.4 - 1386

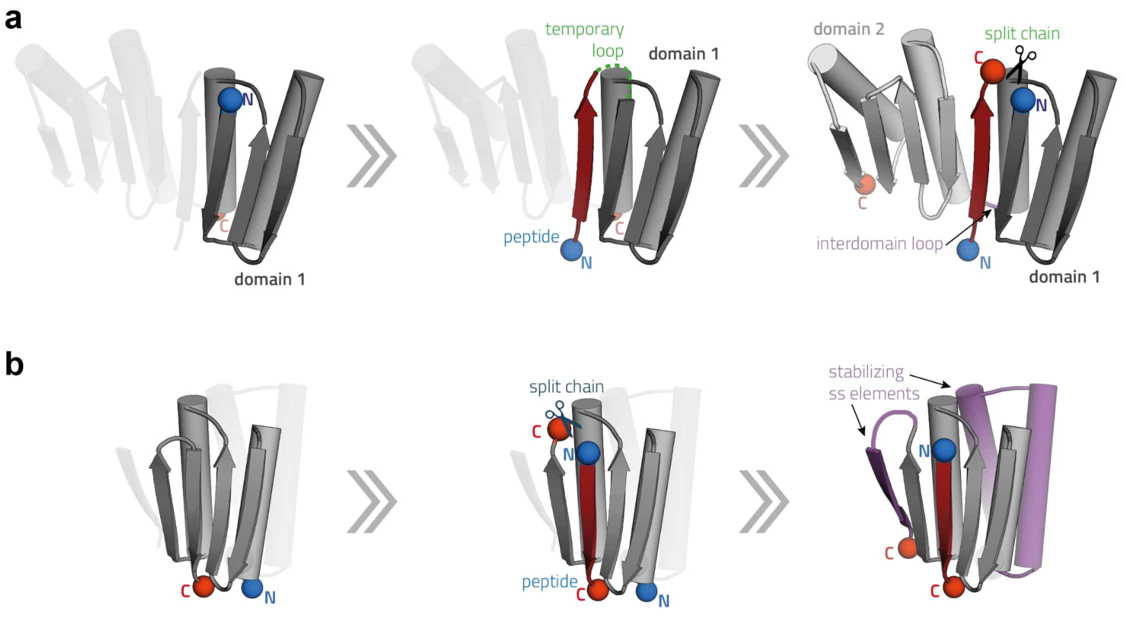

Supplementary Fig. 1. Design approach beta peptide binders. **a,** In approach 1 previously published (Koepnick et al. 2019) scaffold 2003285_0000 (gray, domain 1) was extended with a strand (dark red) and a globular alpha/beta domain (light gray) using blueprint based backbone building. The peptide was generated by introducing chain breaks. Loop closure between the C-terminus of domain 1 and N-terminus of domain 2 yields a single chain beta peptide binder in which the peptide complements the large beta sheet encompassing domains 1 and 2. **b,** In approach 2 a chain break was introduced in the connecting loop between strand 3 and 4 of the previously published scaffold 2003333_0006 (Koepnick et al. 2019) (gray) to generate the peptide (dark red). Stabilizing secondary structure elements (purple) were built using blueprint based backbone building at the N and C terminus of the scaffold.

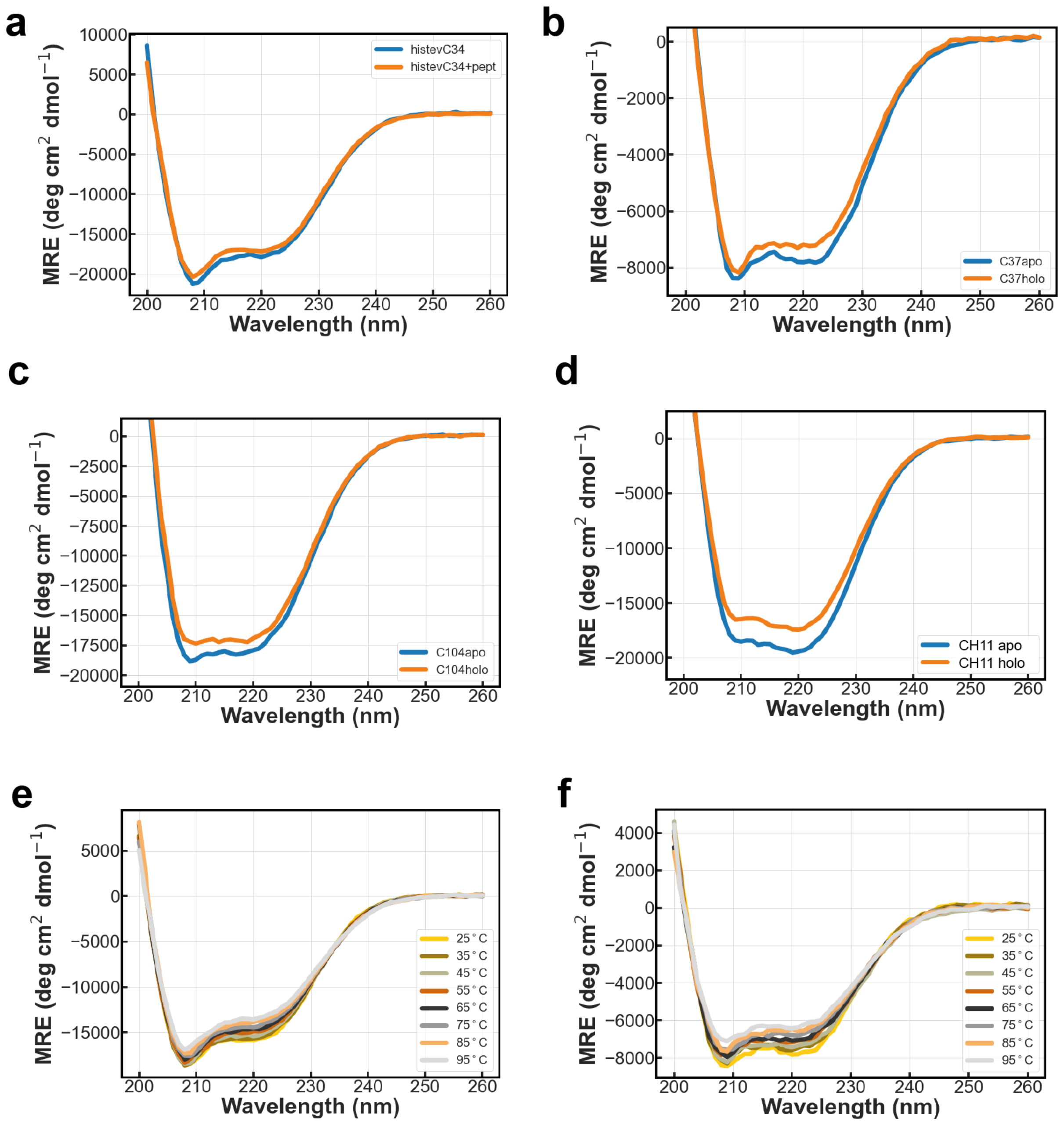

Supplementary Fig. 2. Circular dichroism spectroscopy of designs. a-d Circular dichroism (CD) spectrum at 25°C of various binders with (blue) and without peptide (orange). **e,** CD spectra of C34 at different temperatures. **f,** CD spectra of C37 at different temperatures.

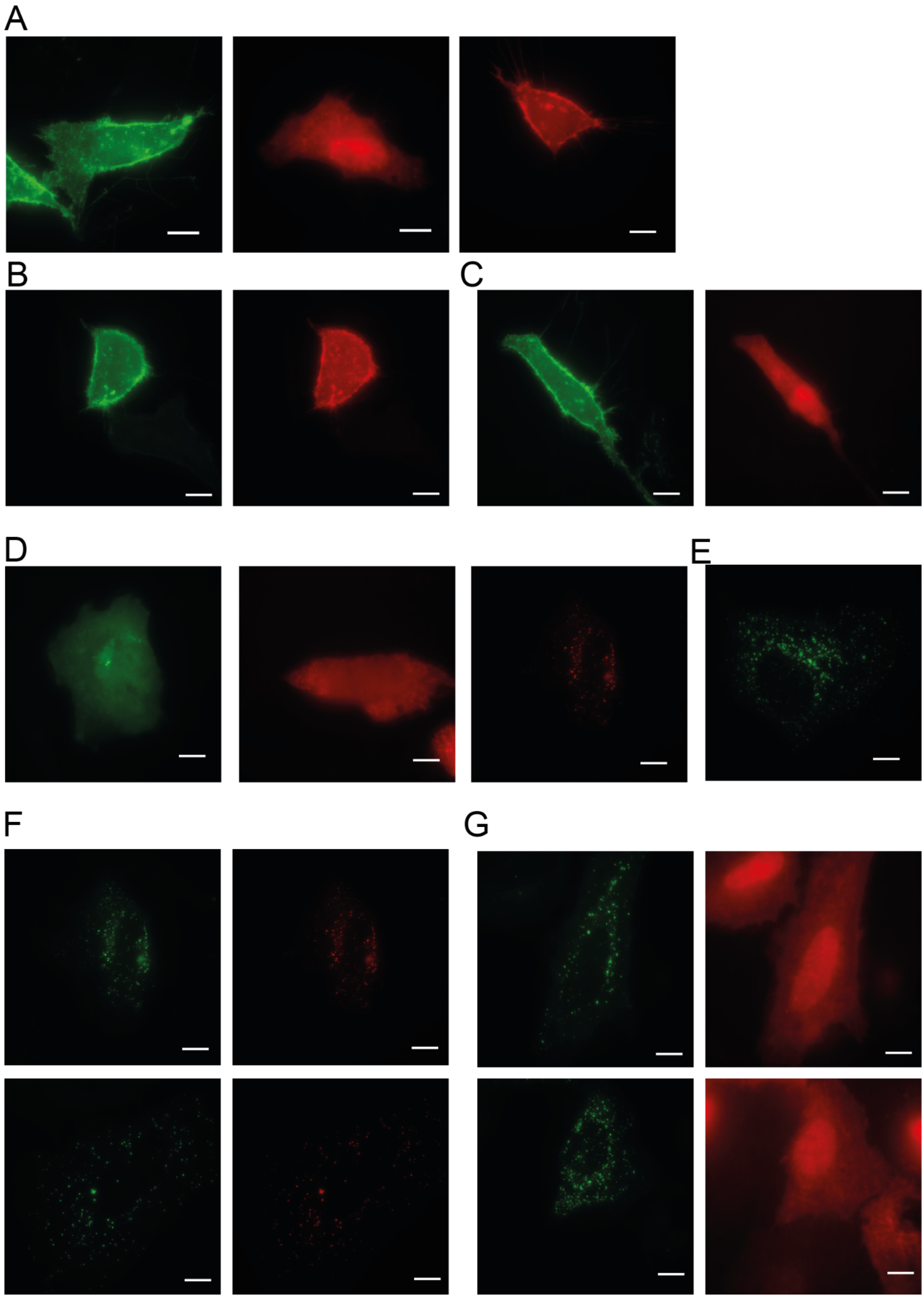

Supplementary Fig. 3. Fluorescent microscopy peptide-binder localization CH15.1. in HeLa cells. a, Uncropped views Fig. 3a. **b,** Alternative view membrane localization. **c,** When F5K/L16K double mutant intended to disrupt binding is introduced to the peptide of CH15.1, CH15.1 binder fused to mScarlet (red channel) does not localize to the membrane anymore.

d, Uncropped views Fig. 3b. **e,** View of a cell co-transfected with C5 and C2 component but without hairpin binder. **f,** Alternative views of localization of CH15.1 mScarlet fusion to the two component GFP puncta. **g,** Two views showing that when the F5K/L16K double mutant is introduced to the peptide of CH15.1 the binder does not localize to the puncta anymore (red channels) even though the puncta still form (green channels). Scale bars 10 μm. Results were reproduced in two independent experiments.

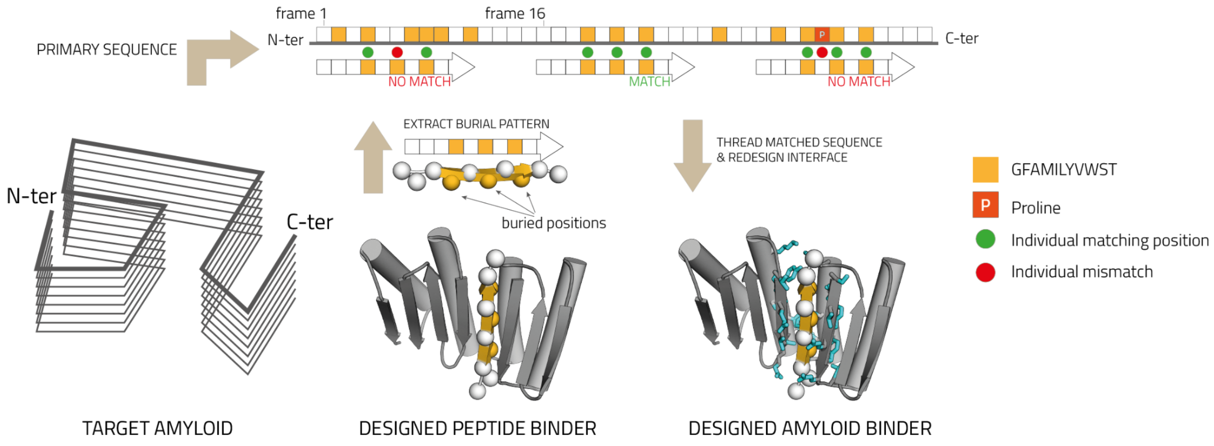

Supplementary Fig. 4. Amyloidogenic sequence docking. The designed peptides are optimized to only harbor GFAMILYVWST residues at the buried positions (yellow); charged residues cannot be accommodated at buried positions due to the high chance of burying a polar residue that cannot be satisfied by complementary side chains on the scaffold. To identify stretches of sequence present in amyloidogenic proteins that can be accommodated in a beta strand conformation in the binding pockets of the designs (fig 2a), the burial pattern of the peptides (middle) are matched to the primary sequence of the amyloidogenic protein (top). Because prolines disrupt beta conformation they are only allowed at termini. When a match is found the original peptide sequence is mutated to the matched sequence from the amyloid protein (threading) and docked back into the scaffold binding pocket followed by redesign of the scaffold interface residues (cyan sticks) to optimize interactions to the amyloidogenic sequence (right).

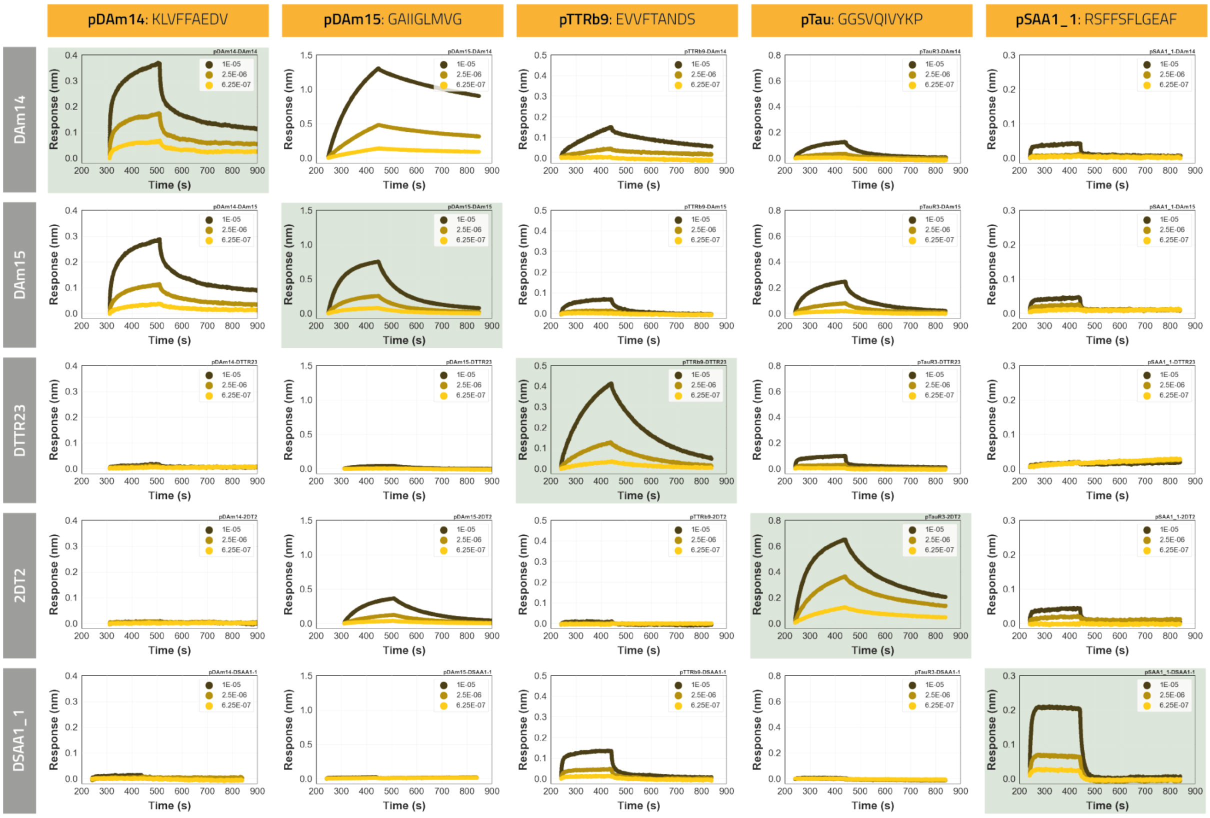

Supplementary Fig. 5. Specificity profile amyloidogenic peptide binders in BLI. Biotinylated peptides were immobilized onto octet streptavidin biosensors at equal densities and incubated with all binders in separate experiments at three concentrations (10, 2.5 and 0.625 µM). The designed on-target interactions are indicated with a light green background.

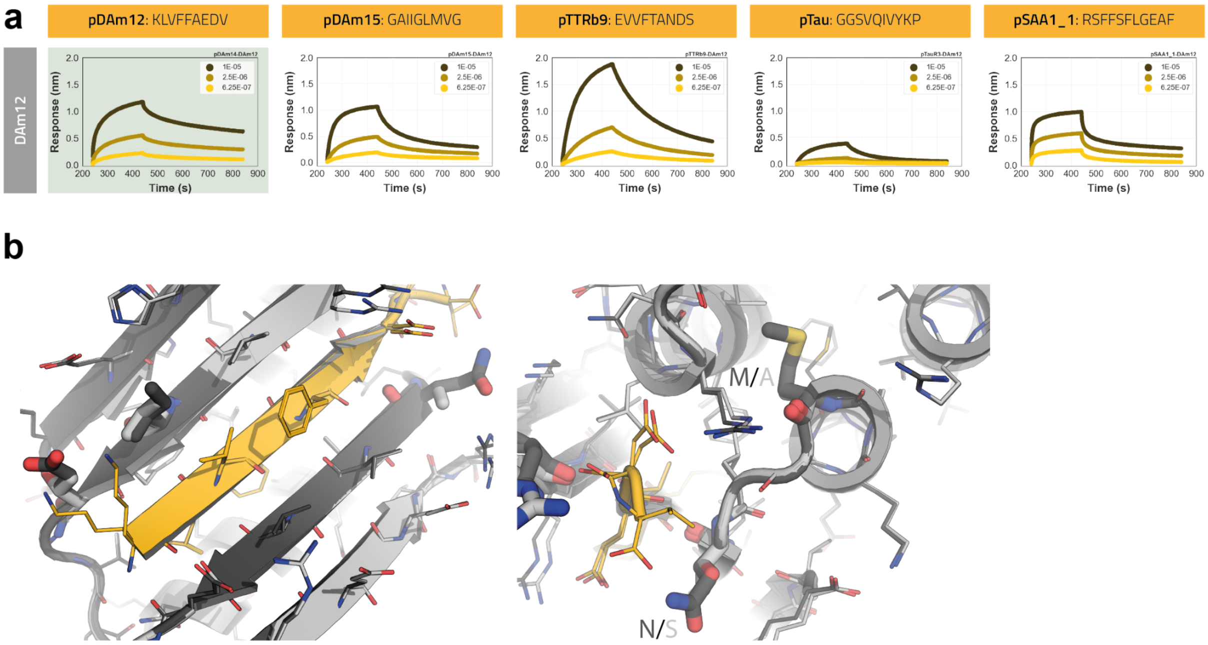

Supplementary Fig. 6. Binding characterization DAm12. a, DAm12 titration (10, 2.5 and 0.625 µM) against all immobilized amyloidogenic peptides in BLI. pDAm12 and pDAm14 are the same peptides. **b,** DAm12 and 14 are similar but have 4-fold difference in Aβ42 monomer binding in MDS assays. Interface close up views that highlight the interface of DAm12 (dark gray) and DAm14 (light gray). Differences in side chains are depicted in thicker sticks.
