# Supplementary figures and images for: Design of amyloidogenic peptide traps
Source: Nat Chem Biol. 2024 Mar 19;20(8):981–90. doi: 10.1038/s41589-024-01578-5 (PMC11288891; doi:10.1038/s41589-024-01578-5)

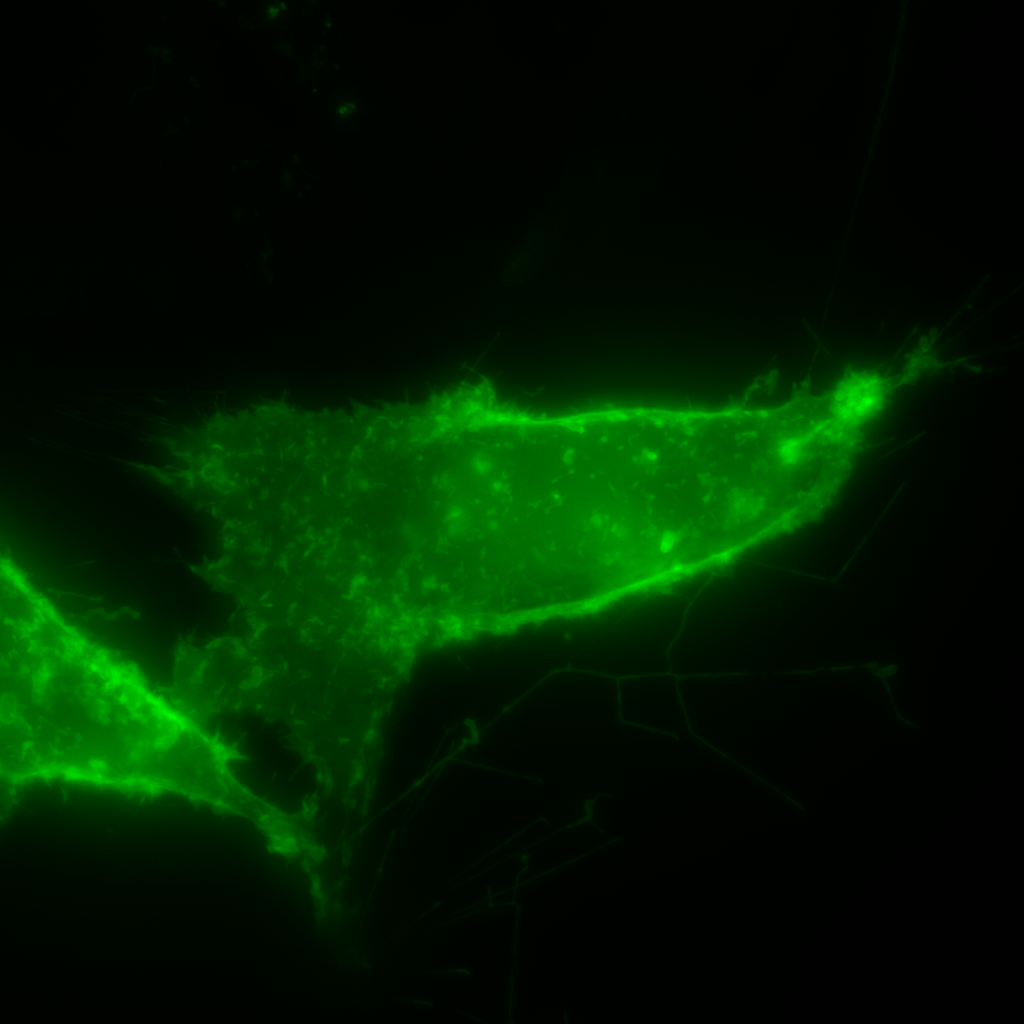

Supplement: Supplementary file 7 — Uncropped image. [file 41589_2024_1578_MOESM7_ESM.zip › green_MAX_condition_1_1514_006.png]

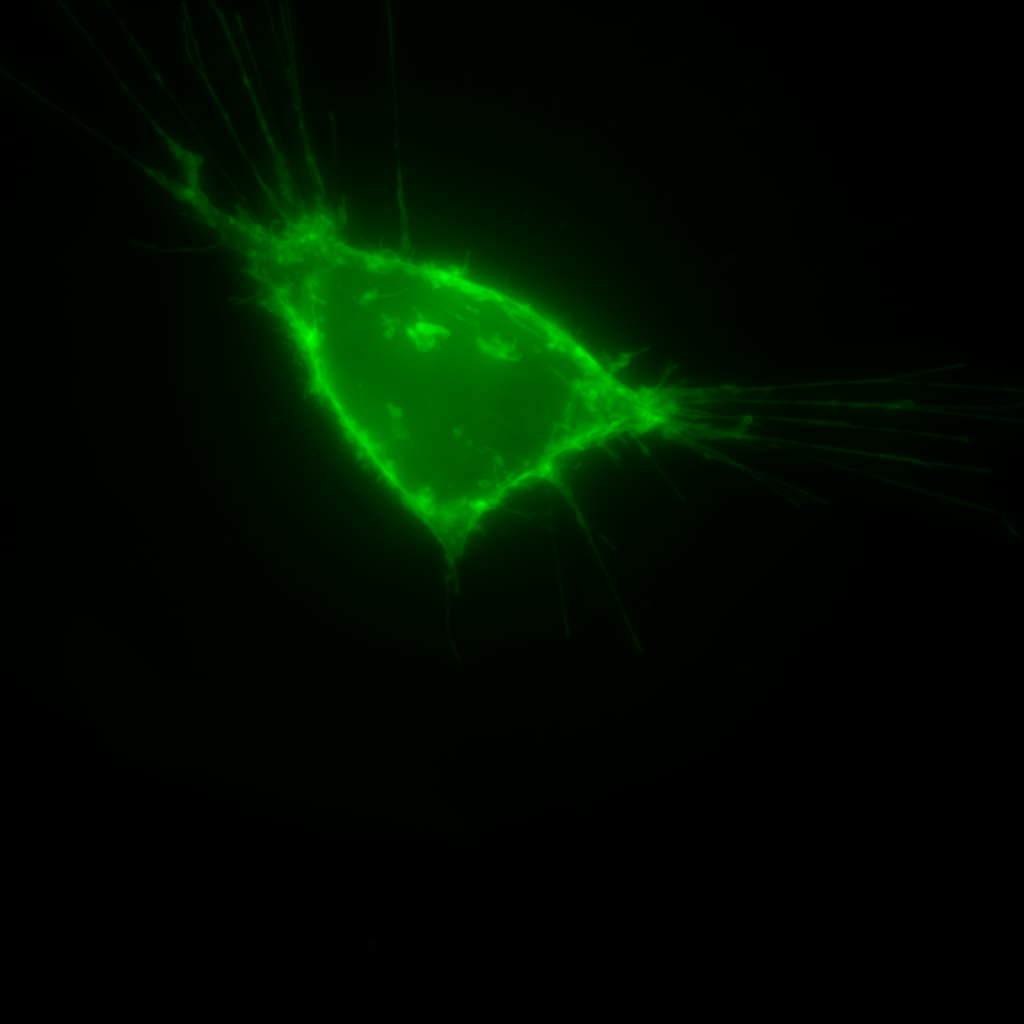

Supplement: Supplementary file 7 — Uncropped image. [file 41589_2024_1578_MOESM7_ESM.zip › green_MAX_condition_4_1514_035.png]

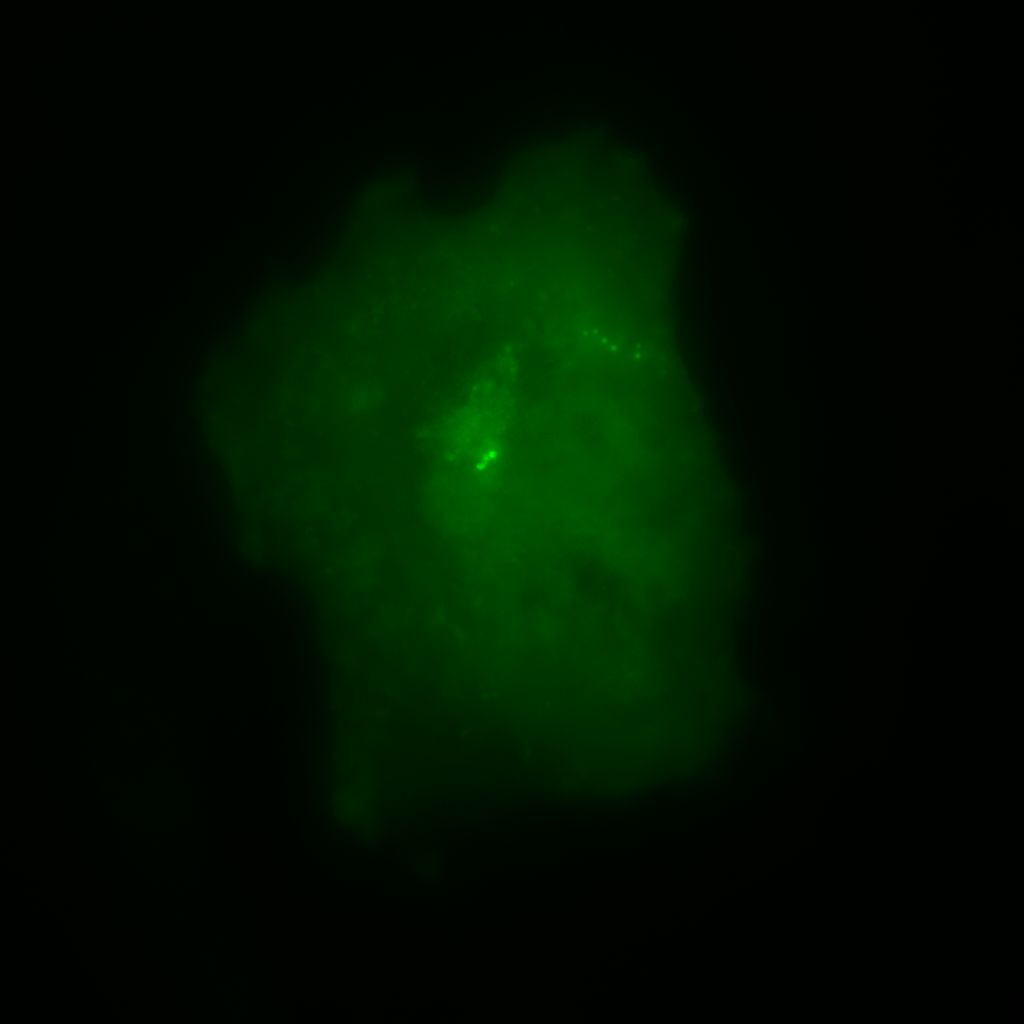

Supplement: Supplementary file 7 — Uncropped image. [file 41589_2024_1578_MOESM7_ESM.zip › green_MAX_condition_6_1514_045.png]

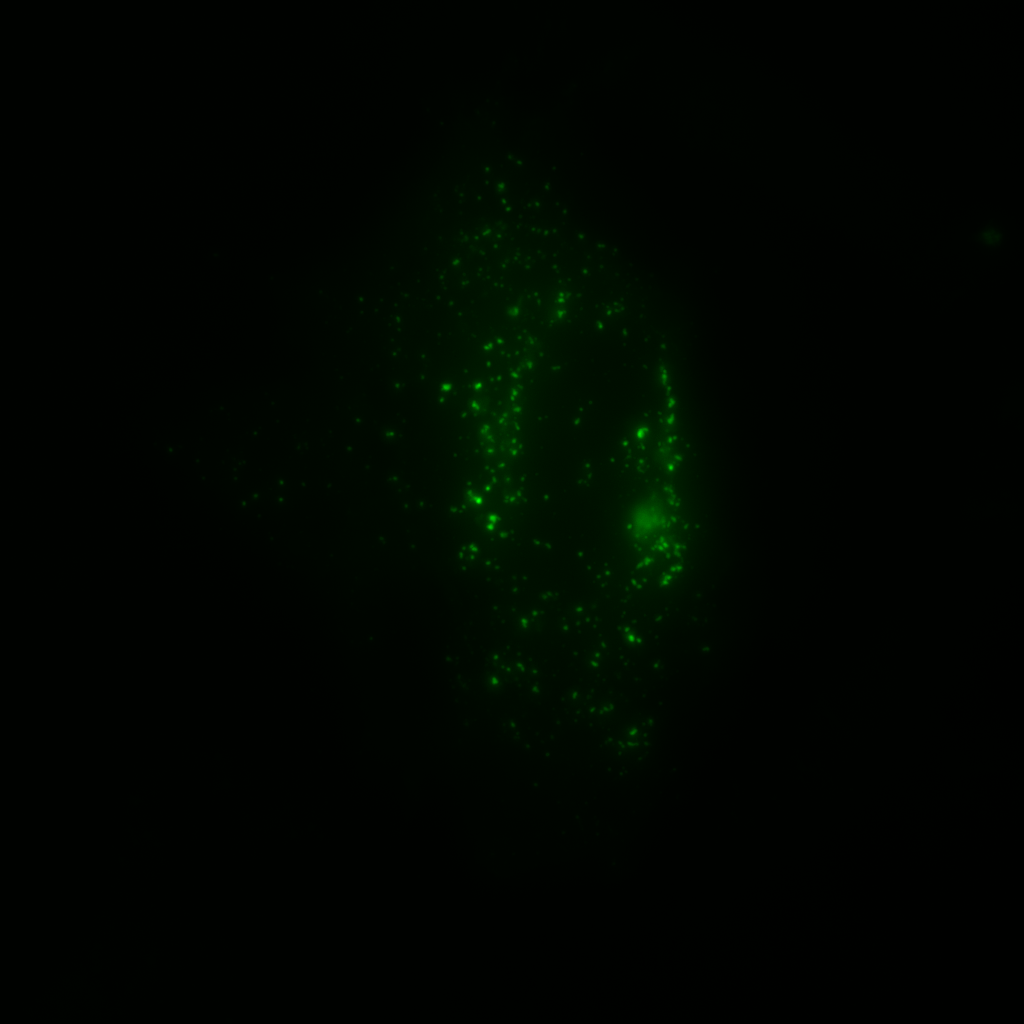

Supplement: Supplementary file 7 — Uncropped image. [file 41589_2024_1578_MOESM7_ESM.zip › green_MAX_condition_8_1514_070.png]

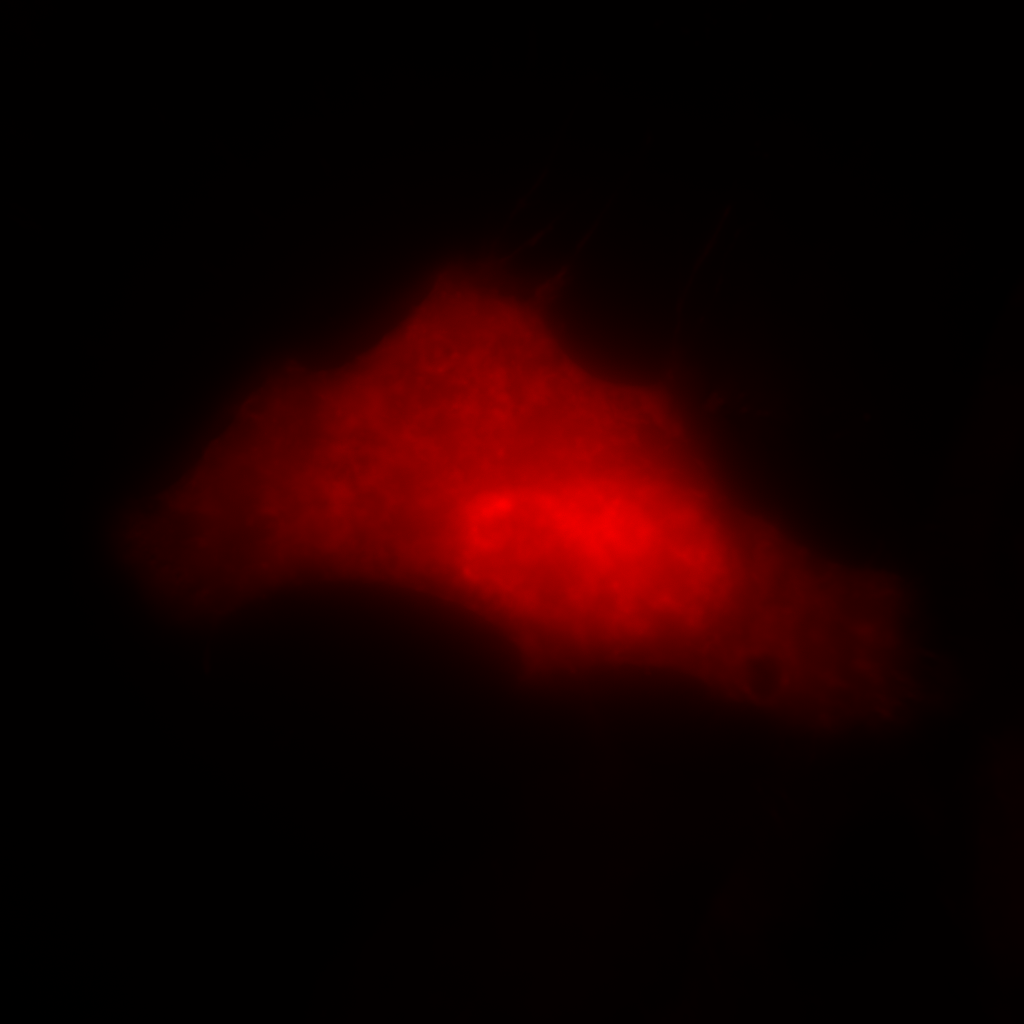

Supplement: Supplementary file 7 — Uncropped image. [file 41589_2024_1578_MOESM7_ESM.zip › red_MAX_condition_3_1514_010.png]

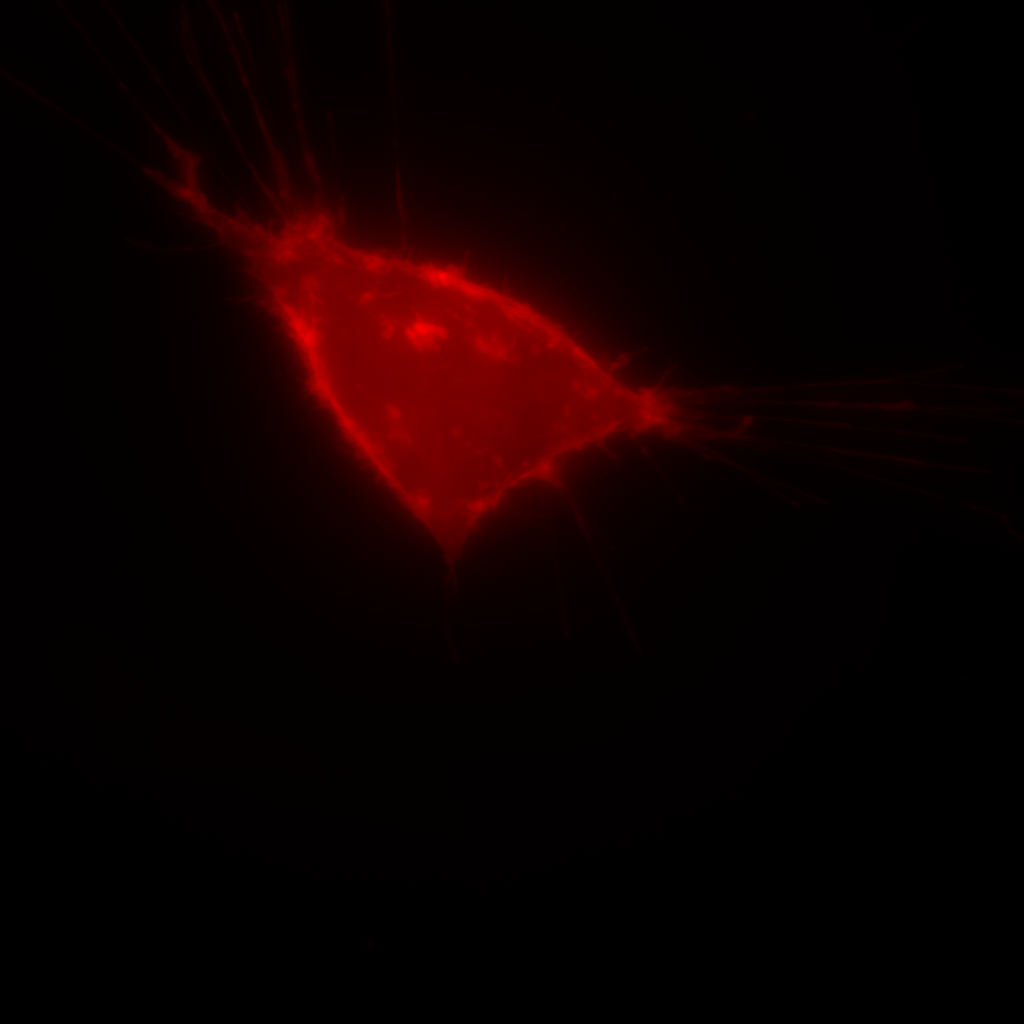

Supplement: Supplementary file 7 — Uncropped image. [file 41589_2024_1578_MOESM7_ESM.zip › red_MAX_condition_4_1514_035.png]

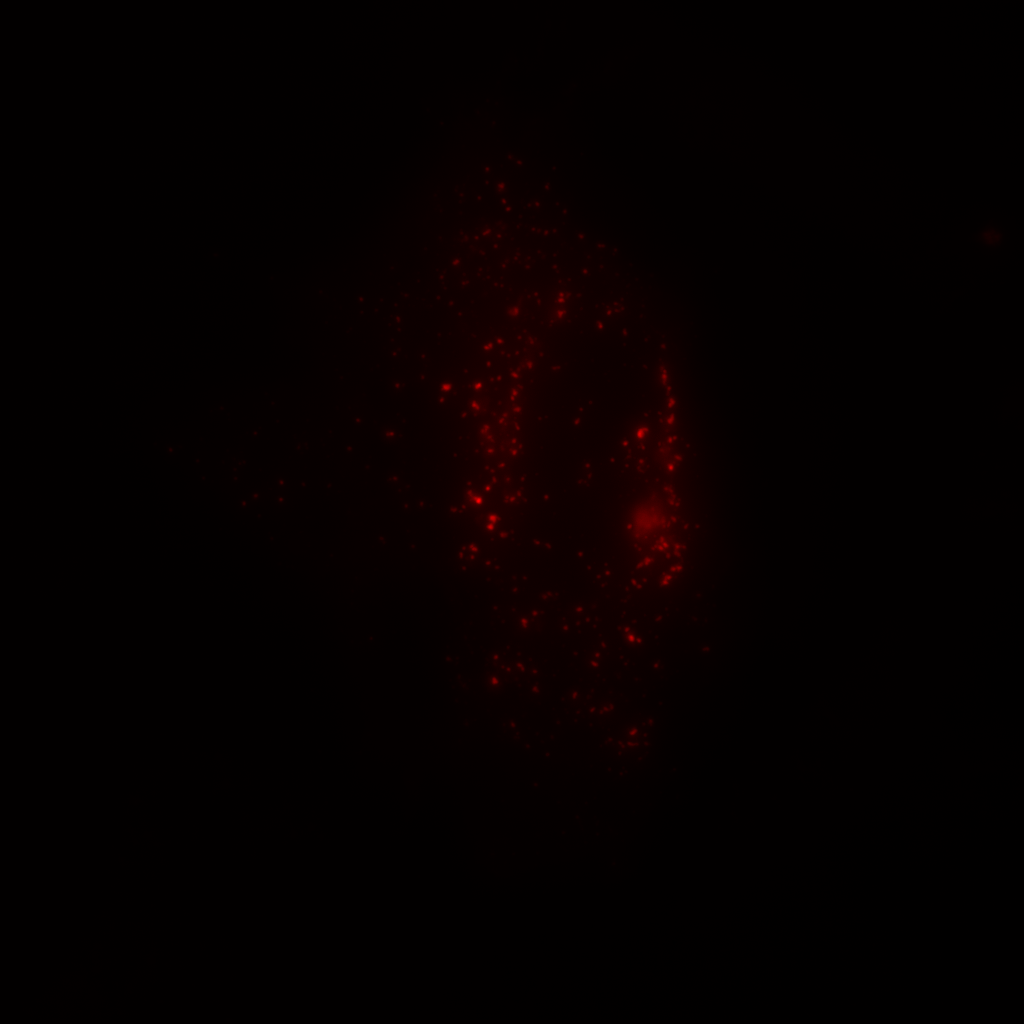

Supplement: Supplementary file 7 — Uncropped image. [file 41589_2024_1578_MOESM7_ESM.zip › red_MAX_condition_8_1514_070.png]

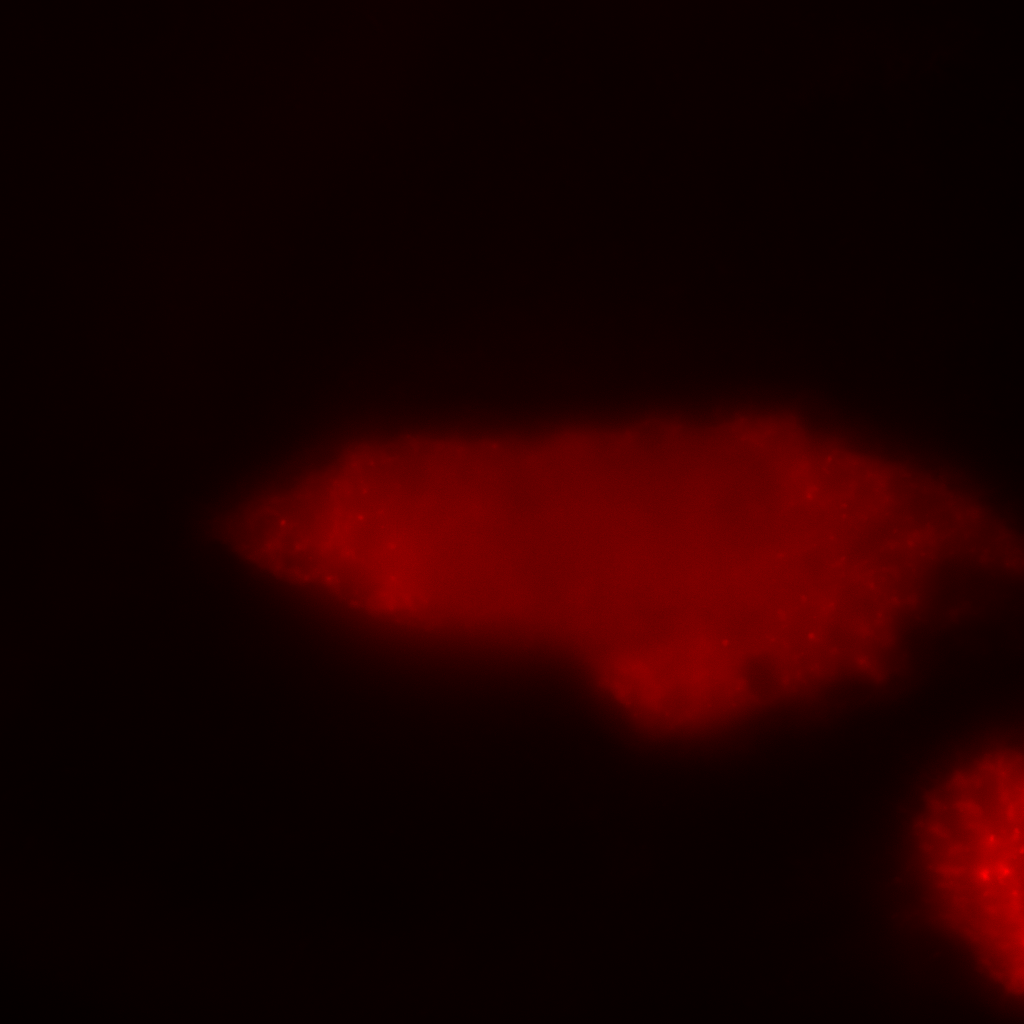

Supplement: Supplementary file 7 — Uncropped image. [file 41589_2024_1578_MOESM7_ESM.zip › red_MAX_condition_9_1514_083.png]
